# Mild cognitive impairment in Parkinson's disease: current view

**DOI:** 10.3389/fcogn.2024.1369538

**Published:** 2024-04-05

**Authors:** Kurt A. Jellinger

**Affiliations:** Institute of Clinical Neurobiology, Vienna, Austria

**Keywords:** Parkinson's disease, mild cognitive impairment, neuroimaging, neuronal network dysfunctions, neuropathology, biomarkers

## Abstract

Parkinson's disease (PD), the most common motor movement disorder and second most common neurodegenerative disorder after Alzheimer's disease (AD), is often preceded by a period of mild cognitive impairment (MCI), which is associated with impairment of a variety of cognitive domains including executive function, attention, visuospatial abilities and memory. MCI, a risk factor for developing dementia, affects around 30% of *de novo* PD patients and can increase to 75% after more than 10 years. While 30–40% remain in the MCI state, up to 60% will convert to dementia. Characteristic findings are slowing of EEG rhythms, frontotemporal hypoperfusion, decreased functional connectivity in the default mode and attentional networks, prefrontal and basal-ganglia-cortical circuits, which often manifests prior to clinical symptoms and overt brain atrophy. The heterogeneity of cognitive phenotypes suggests that a common neurodegenerative process affects multiple functional neuronal networks and neuromodulatory systems that may be superimposed by Lewy body and Alzheimer's-related or other co-pathologies. Sparse neuropathological data for PD-MCI revealed a heterogenous picture with various morphological changes similar to MCI in other diseases. This review highlights the essential epidemiological, clinical, neuroimaging and morphological changes in PD-MCI, available biomarkers, and discusses the heterogenous pathobiological mechanisms involved in its development. In view of its complex pathogenesis, well-designed longitudinal clinico-pathological studies are warranted to clarify the alterations leading to MCI in PD, which may be supported by fluid and neuroimaging biomarkers as a basis for early diagnosis and future adequate treatment modalities of this debilitating disorder.

## 1 Introduction

Parkinson's disease (PD), one of the most common neurodegenerative diseases with a rising global prevalence (Wolff et al., 2023), is caused by the widespread deposition of misfolded α-synuclein (αSyn), a neuronal protein forming intraneuronal Lewy bodies (LBs) and Lewy neurites, the morphological hallmarks of PD and related synucleinopathies. These changes cause progressive degeneration not only of the dopaminergic striatonigral system, responsible for the core motor symptoms, but of many other neuronal systems and organs. The resulting biochemical deficits are responsible for the heterogeneous spectrum of motor and non-motor symptoms of PD that contribute to the overall disease burden of this multisystem/multiorgan disorder (Dickson et al., 2009; Jellinger, 2012, 2022; Poewe et al., 2017). Its complex pathogenic pathways including oxidative stress, abnormal proteasome management, mitochondrial, synapse and lysosomal dysfunction, neuroinflammation, and other molecular processes, related to spreading of abnormal proteins in cortical and subcortical regions with subsequent dysregulation of multiple neuromodulator systems, are well-documented (Zaman et al., 2021; Koros et al., 2022; Calabresi et al., 2023; Hussein et al., 2023; Ye et al., 2023).

Cognitive impairment (CI) has been increasingly recognized as an integral and most disabling non-motor symptom of PD that shows a broad spectrum from subjective cognitive decline (SCD) or subjective cognitive complaints (SCCs) and mild cognitive impairment (MCI) to full-blown Parkinson's disease dementia (PDD). They can be present decades before manifest motor symptoms, i.e., in the pre-clinical phase of PD (Jessen et al., 2014; Weil et al., 2018; Flores-Torres et al., 2023). SCD in PD is a self-perceived decline in cognitive abilities with normal age-, sex-, and education-adjusted performance on standardized cognitive tests indicating adequate cognitive function (Oedekoven et al., 2022), although there is currently no consensus of clinical criteria for SCD in PD (Oedekoven et al., 2022; Huang J. et al., 2023). Since it is already associated with metabolic changes in multiple cortical regions indicating early aberrant pathological changes (Ophey et al., 2022), SCD is considered an increasing risk of developing dementia (Pike et al., 2022). SCCs refer to self-perceived cognitive decline in cognitive normal individuals and are considered a preclinical sign of subsequent CI. SCCs as measured by the Cognitive Complaints Interview (CCI) are strongly correlated with objective cognitive performance; the CCI score increases with age and is inversely correlated with cognitive performance (Hong et al., 2018). Since there are no definite guidelines for PD-SCD (and -SCCs), researchers have classified it differently, and some defined it as subjective CI without objective cognitive decline but poor performance in action naming (Galtier et al., 2023) and an indicator of subsequent CI (Purri et al., 2020; Hong and Lee, 2023), although they are very common in PD and increase with duration of PD (Hong et al., 2018). Inclusion of SCCs in PD-MCI criteria may strengthen the ability to detect persons with risk factor for future cognitive decline in early PD (Jones et al., 2023), although the prevalence and risk factors for SCCs have been found to be distinct in PD with and without MCI, suggesting that SCCs in early PD with different cognitive status may have different pathogenicity (Pan et al., 2021).

The concept of MCI, described in Prichard (1837), encompasses an intermediate stage between normal cognitive ability and dementia, in which SCD and objective CI are present, while activities of daily life are preserved (Petersen et al., 1999; Albert et al., 2011). MCI, representing early clinical features of cognitive disorders, such as Alzheimer's disease (AD) or other dementia disorders (Morris et al., 2001; Winblad et al., 2004; Gauthier et al., 2006; Jicha et al., 2010), describes a gradual decline of cognitive abilities affecting single or multiple cognitive domains on complex tasks (Petersen et al., 2009; Litvan et al., 2012; Pedersen et al., 2017). MCI affecting newly diagnosed untreated (*de novo*) and juvenile PD patients may be associated with subtle changes of cognitive function that are not recognized by patients, family members or even clinicians (Bougea et al., 2019). These changes may occur in pre-symptomatic stages of PD (Fengler et al., 2017), preceding the onset of severe CI/dementia by up to 20 years, as well as at the time of diagnosis or during the whole disease process. PD-MCI has an increased risk for conversion to PDD (Janvin et al., 2006; Williams-Gray et al., 2007; Litvan et al., 2012; Nicoletti et al., 2019; Giil and Aarsland, 2020; Wallace et al., 2022). The heterogeneity of PD-MCI may reflect the underlying pathogenic mechanisms as well as the impact of co-morbidities, such as AD-related changes, that are incompletely understood. The present article is intended to review the currently available clinical, epidemiological, neuroimaging data and biomarkers concerning PD-MCI, based on literature research in PubMed and Google Scholar until January 2024 and personal experience in a large number of autopsy-proven PD cases with and without cognitive dysfunctions.

## 2 Diagnostic criteria for PD-MCI

PD-MCI has a high variability in severity, rate of progression and type of affected cognitive domains (Aarsland et al., 2021; Koros et al., 2022; Carceles-Cordon et al., 2023). Furthermore, there is a heterogeneity of cognitive symptoms in various PD subtypes, patients with non-tremor dominant motor symptoms and with depression developing more often and more severe MCI (Tremblay et al., 2013). Although it can manifest as deficits in nearly all cognitive domains, executive dysfunction is most pronounced (Zgaljardic et al., 2003; Kudlicka et al., 2011) that may manifest earlier and more frequent than previously appreciated (Tröster, 2008). The Movement Disorder Society Task Force (MDS-TF) has delineated diagnostic criteria for PD-MCI (Litvan et al., 2012; Goldman et al., 2015). They are as follows: (1) a diagnosis of PD, (2) a gradual decline of cognitive ability in the level I category based on a brief cognitive assessment or on level II category based on a more comprehensive neuropsychological assessment including at least two tests for each of five cognitive domains (attention, executive function/EF/, language memory, visuospatial function, and memory), and (3) complete functional independence. Impairment in neuropsychological tests is defined per one to two standard deviations below the appropriate norms, a significant decrease in the serial cognitive tests, or a significant decrease from the estimated premorbid levels. PD-MCI patients according to the level II criteria show impairment in at least two tests within one single cognitive domain or different domains. Exclusion criteria are definite PDD, other explanations for Cl and PD-associated co-morbidities. Other definitions for PD-MCI are impairment of at least one cognitive domain, defined as 1.5 times worse performance on *z*-scores, compared with normal controls (Caviness et al., 2007; Aarsland et al., 2010), and deficits in at least four cognitive domains (executive, attention, visuospatial abilities, and memory), being severe enough to interfere with routine functions in everyday live (Svenningsson et al., 2012; Kiesmann et al., 2013). For other criteria see Saredakis et al. (2019). The most frequent phenotypes of MCI in prodromal PD are deficiencies in executive, visuospatial and linguistic tasks and multi-domain amnestic phenotypes (Ciafone et al., 2020), without essential affection of memory (Speelberg et al., 2022). PD-MCI subjects were divided into four subgroups: non-amnestic multiple-domain type (PD-naMCI-MuD), non-amnestic single-domain type (PD-naMCI-SiD), amnestic single-domain type (PD-aMCI-SiD), and amnestic multiple-domain type (PD-aMCI-MuD). Differences between PD-MCI and PD-NC with respect to age, age at onset, years of education, and motor symptom severity were significant. In contrast to the population as a whole, where aMCI is the most common subtype, in PD naMCI is dominant, with prominent executive and attention dysfunctions (Yarnall et al., 2013), whereas in another group PD-aMCI was most common (Vasconcellos et al., 2019). In a large US sample, the most common subtype was PD-naMCI-SiD (47.7%), followed by aMCI-MuD (24.2%), aMCI-SiD (18.8%), and naMCI-MuD (9.5%; Goldman et al., 2012a). In others, the single-domain type was the largest PD-MCI subgroup (52.83%), and within single domain impairment, non-amnestic is more common than the amnestic one (Litvan et al., 2011). Another study included 39.4% naMCI-SD, 30.5% aMCI-MuD, 23.4% naMCI-MuD, and 6.7% aMCI-SiD subtypes, EFs being most frequently impaired (Kalbe et al., 2016). A recent meta-analysis confirmed a higher frequency of multiple domain MCI subtype (Baiano et al., 2020). The most common PD-aMCI subtype was associated with a lower quality of life compared with the non-amnestic group (Vasconcellos et al., 2019). Memory and EF impairment were most frequent (22.64 and 20.75%, respectively). The overall cognitive function in aMCI-MuD was significantly worse compared with the SiD type (Nie et al., 2019). These lesions were more pronounced in early stages of PD, executive dysfunction being most pronounced. Working memory appeared to decline slightly faster in early PD compared to healthy controls (HCs), while other domains remained similar without considerable changes (Turner et al., 2023). PD-MCI patients had greater subjective cognitive and communicative problems than PD-NC ones (Jaramillo-Jimenez et al., 2023). Basic PD-MCI in addition to diffuse impairment of EF, involved visuospatial ability and construction, language, working memory, slowed processing speed and verbal fluency, as well as impaired attention, daytime sleepiness (Ciafone et al., 2020). Clear deficits in EF and attention, observed 6 years before diagnosis, were predictive of poorer outcomes at follow-up (Williams-Gray et al., 2007). A diagnosis of prodromal PD should be considered in individuals with MCI who present with prominent executive, attentional and visuospatial deficits, worse verbal fluency and less memory impairment (Yoon et al., 2014; Brønnick et al., 2016). A meta-analysis of five longitudinal studies of PD-MCI reported a predominant involvement of visuospatial skills and visual memory as well as longitudinal executive dysfunction (Wallace et al., 2022). Heterogeneous cognitive subgroups warrant further investigation to advance effective evaluation (Yang et al., 2023).

PD-MCI patients show slower non-decision times than controls, which is negatively correlated with visuospatial performance, but they have no impairment of information processing ability. Pausing before word production is associated with PD-MCI, and these pauses are correlated with Montreal Cognitive Assessment (MoCA) score but not with motor severity (Andrade et al., 2023). Moreover, PD-MCI is associated with gait dysfunction (Morris et al., 2017), characterized by changes in mean velocity, step and circle length and other features (Amboni et al., 2022; Russo et al., 2023). Other changes in individuals with PD and MCI are sleep problems and reduced proprioceptive and olfactory sensitivities, which are related to disease severity (Li et al., 2023).

## 3 Epidemiology

### 3.1 PD-SCD and MCI

Due to the inconsistent classification of SCD, its reported frequency varied considerably between 25 and 85% (Dujardin et al., 2010; Hong et al., 2012; Erro et al., 2014; Barbosa et al., 2019; Galtier et al., 2019; Pan et al., 2021; Siciliano et al., 2021; Huang J. et al., 2023). In a cohort of *de novo* PD patients, 42.3% reported SCCs and 53.5% were diagnosed MCI. The prevalence of SCD in PD-MCI and PD-normal cognition (NC) participants was 30.3 and 12.1%, respectively. The frequency and risk factor of SCD were distinct in PD with and without MCI, which suggested that SCD in early PD with different cognitive status appears to have different pathogenicity (Pan et al., 2021). PD-MCI rates were consistently higher (16.5–19.1%) across the 5 years when SCD was not included in the diagnostic criteria as opposed when SCD was included (4.4–11.0%). Thus, PD-MCI + SCD experienced greater cognitive deficits and had significantly higher levels of cerebrospinal fluid (CSF) tau and phophorylated tau (p-tau) proteins. Even in PD patients with SCCs without objective CI, later cognitive deterioration could not be excluded (Siciliano et al., 2024). Inclusion of SCD in PD-MCI criteria in *de novo* PD patients may strengthen the ability to detect individuals at risk for future cognitive decline (Jones et al., 2023).

### 3.2 PD-MCI

MCI, a transitional stage between normal cognition, SCD and dementia, that can occur at any stage of PD, has a frequency ranging from 19 to 62%, with an estimated point prevalence of 30% (Caviness et al., 2007; Williams-Gray et al., 2007; Dalrymple-Alford et al., 2011; Erro et al., 2012; Weintraub et al., 2012; Jellinger, 2013; Galtier et al., 2016, 2019; Monastero et al., 2018; Nicoletti et al., 2019; Nie et al., 2019; Aarsland et al., 2021; Yang et al., 2023; Zhang P. et al., 2023). In an earlier review it ranged from 18.9 to 55% (Goldman and Litvan, 2011). These deviations can be explained by differences in the PD-MCI diagnostic citation selection bias and number of tests used. About 20–30% have at least mild cognitive changes at the time of PD diagnosis (Poletti et al., 2012) increasing to 40–50% after 5 years follow-up (Domellöf et al., 2015) and to 75% after survival of more than 10 years (Hely et al., 2008; Brønnick et al., 2016). About 30% of *de novo* PD patients complain of mild memory problems and are likely to develop MCI after 2 years follow-up (Purri et al., 2020), but other factors, such as depression and anxiety, may contribute to progression of mild cognitive deficits (Bernard et al., 2020; Petkus et al., 2020). A recent meta-analysis reported a pooled PD-MCI prevalence of 40% (Baiano et al., 2020), while its estimated prevalence in the general population (age 60–90 years) ranged between 3 and 19% (Ritchie, 2004) or 16 and 20% (Roberts and Knopman, 2013). The frequency of MCI in young-onset PD patients is smaller (5–10%) compared to older ones (22–25%; Santos-García et al., 2023). Women experience a worse global cognitive decline in the prodromal phase of PD (Flores-Torres et al., 2023). In a large cohort of early PD, between 22.8 and 53.7% fulfilled the diagnostic criteria for PD-MCI (Wang et al., 2014; Huang et al., 2020; Yang et al., 2023). The prevalence of MCI was ~40 and 32% among newly diagnosed PD patients (Monastero et al., 2018). More recent studies reported 69.3% PD-MCI, 16% PD-NC and 14.55% PDD (Sousa and Brucki, 2023), or 34% PD-MCI, 77.8% of which impaired in multiple cognitive domains (Yang et al., 2023). Between 20 and 57% of PD patients were affected by MCI within the first 3–5 years after diagnosis (Caviness et al., 2007; Meireles and Massano, 2012). More sophisticated neuropsychological assessment (MDS-TF criteria level II) detected MCI in 60.5% of PD patients, but only 23.3% when only brief assessment was performed; multiple domain impairment was most frequent (96.2%; Galtier et al., 2016). Based on the MoCA, 56.5% of PD patients had MCI, based on Parkinson's disease cognitive rating scale (PDCRS) only 37%. From the MCI group, 77.3% had severe executive dysfunction (Samat et al., 2017). In a study, classifying PD-MCI according to the level II MDS-TF criteria, 34% met criteria for MCI (significantly more males), with impairments in multiple cognitive domains in 77.8% (Yang et al., 2023). Another study diagnosed 43% as PD-MCI using the level II MDS-TF criteria (Uysal-Cantürk et al., 2018). A multicenter analysis revealed an average prevalence of MCI of 25.0%, 18.9% in an incidental, untreated cohort, and 39% in advanced PD. In the Parkinson's disease cognitive impairment study (PACOS), PD-MCI was diagnosed in 31.7% after disease duration <1 year, and in 39.6% after a mean duration of 3.8 ± 4.6 years. Amnestic MCI multidomain phenotype was the most frequent (39.1%) of the overall sample and 43.9% in newly diagnosed PD. The prevalence of MCI was ~40 and 32% among newly diagnosed PD patients (Monastero et al., 2018). In a US sample 95% were diagnosed multiple-domain PD-MCI and a range of CIs was noted with overlap of several factors (Cholerton et al., 2014). More recent studies reported 69.3% PD-MCI, 16% PD-NC, and 14.7% PDD (Sousa and Brucki, 2023). Validation studies have reinforced the hypothesis that PD-MCI is more frequent than previous studies showed without applying MDS criteria and confirmed that it is a risk factor for the onset of dementia (Galtier et al., 2016).

### 3.3 MCI converters

A three-stage clinical sequence related to cognition has been proposed for PD patients, with SCD as the prodromal phase, followed by MCI and finally leading to dementia (Jones et al., 2021; Yoo et al., 2021). Follow-up studies showed a higher risk of developing MCI for patients with PD-SCD compared to those without (Purri et al., 2020; Hong and Lee, 2023). Lower baseline attention and language scores were associated with progression to MCI, whereas higher baseline scores in all cognitive domains except EFs were associated with clinical and psychometric reversion to “normal” cognition, although an increased risk of developing CI persisted in these patients (Gasca-Salas et al., 2023). PD-MCI converters showed more severe cognitive deficits in frontal EFs (Chung et al., 2017), immediate verbal memory and visual recognition memory compared to non-converters (Lee et al., 2014). The pooled conversion rate from PD-NC to PD-MCI over follow-up times ranging from one to 16 years was 28% (95% CI 24–33%). Among PD-NC individuals, within 3 years 25% (95% CI 20–30%) converted to MCI and 2% (95% CI 1–7%) to PDD, while 28% (95% CI 20–34%) across all folow-up periods reverted back to normal cognitive functions. For studies with follow-up periods over 3 years, the conversion rate increased to 29% (95% CI 22–37%). The average reversion rate from PD-MCI to PD-NC with follow-up times from one to 6 years was 24% (95% CI 17–34%) and with follow-up times under 3 years, the rate was 28% (95% CI 20–37%). Across all follow-ups, using level I criteria, 35% reverted to PD-NC from PD-MCI as compared to 15% using level II criteria (Saredakis et al., 2019). Between 25.4% and 57% of MCI cases converted to PDD at follow-up (Galtier et al., 2016; Nicoletti et al., 2019). Others reported a conversion rate between 38 and 51% (Camicioli and McDermott, 2018). Fifty-nine percent of PD patients with persistent MCI within 1 year developed PDD (Pedersen et al., 2017), and 62% after 4-year follow-up (Janvin et al., 2006), compared with 20% in PD-NC patients, and 39–50% after 5-year follow-up (Domellöf et al., 2015; Pedersen et al., 2017). The incidence of progression from PD-MCI to PDD was 98/1,000 person-years, with an annual conversion rate of 11% (Hobson and Meara, 2015), according to others 123.5/1,000 person-years (Nicoletti et al., 2019). The PDD converters had higher frequency of multi-domain MCI and aMCI with poorer performance of frontal executive, memory and language functions (Chung et al., 2019; Yang et al., 2023). As definitions of PD-MCI are increasingly refined and improved, more accurate predictions of the risk of developing dementia in PD based on cognitive profiles of PD-MCI are likely to be developed.

In conclusion, the prevalence of SCD and, in particular, of MCI in PD are significantly higher compared to an age-matched control population, although the frequencies vary considerably due to variable diagnostic definitions and different neuropsychological test modalities.

## 4 Risk factors for PD-MCI

### 4.1 Genetic (risk) factors for PD-MCI

Genetic risk factors of cognitive decline in PD are not well-known to date besides variants in the glucocerebrosidase (GBA) and APOE genes. In PD, more than 100 genes or genetic loci have been identified as risk factors, and most PD cases likely arise from interactions among genetic variants (Ye et al., 2023). As in the pathogenesis of PD, genetic risk factors may play a role for CI. In addition to genes that increase the risk for PD with variable rates of penetrance including SNCA, GBA1, LRRK2, VPS35, PRKN, PINK1, DJ-1, and Parkin (Wise and Alcalay, 2022; Ratan et al., 2024), other risk variants, including PITX3, TMEM106B, SNCA Rep1, COMT, MAPT H1/H1, and APOEε4 have been related to cognitive dysfunction (Wise and Alcalay, 2022). GBA mutations have a negative impact on cognition, whereas LRRK2 may be associated with MCI, the others with a potential effect on cognitive outcome (Fagan and Pihlstrøm, 2017). GBA-related PD (GBA-PD) is associated with faster motor and cognitive decline, especially affecting visuospatial and executive functions (De Michele et al., 2023; Ren et al., 2023), the rs12411216 variant of GBA being associated with PD-MCI (Jiang et al., 2020). Longitudinal cognitive decline in GBA-PD patients was more severe than in LRRK2/GBA cases, which suggests the possibility of an interaction of both alleles, although the biological basis is unclear (Ortega et al., 2021). However, the molecular mechanisms connecting GBA1 gene mutations to increased PD risk remain partly unknown (Dos Santos et al., 2024). APOEε4, a major risk factor for cognitive (and motor) progression in PD (Tan et al., 2021), has an age-dependent effect on decline in global cognition and most cognitive domains (Liu J. Y. et al., 2023). Carriers of GBA and APOEε4 mutations suffered faster cognitive decline with progression to dementia (Real et al., 2023), whereas MAPT and SNCA had no long-term impact on cognitive decline (Szwedo et al., 2022). Aquaporin-4 polymorphism is associated with cognitive performance in PD, subtype rs68006382 with faster progression to MCI, worse performance in semantic fluency and other cognitive domains, which is probably related to alterations of glymphatic efficacy, a sleep-enhanced brain waste clearing system (Fang et al., 2022). Furthermore, a possible role of glial cell line-derived neurotrophic factor (GDNF) for predicting CI in PD has been suggested, which, however, awaits confirmation (Shi et al., 2021). Recent studies investigating the relations of 100 polygenic scores (PGS) to cognitive outcome found four PGS significantly associated with cognitive decline in PD, all associated with general cognitive phenotypes, highlighting the importance of genetic factors for cognitive decline in PD (Faouzi et al., 2024).

### 4.2 Other risk factors for PD-MCI

Other risk factors for PD-MCI are older age, lower education, longer disease duration, higher levodopa equivalent daily dose, severe motor symptoms (akinetic-rigid phenotype), postural instability, gait difficulty, freezing, higher level of apathy, and depression (Baiano et al., 2020). Follow-up of PD patients at one to 5 years showed that PD patients with depression, anxiety and apathy were early to meet the criteria for MCI; those with depression and anxiety showed a progressive decline in all four cognitive domains, while apathy and impulse control disorders were separately associated with a progressive decline in processing speed, attention and memory, respectively (Meng et al., 2023). PD-naMCI performed worse than PD-NC in social cognitive measures, whereas PD-naMCI performed worse than PD-NC in only one subtype, suggesting that subtle changes in social cognition could partly explain future transition into dementia (Maggi et al., 2023).

Cognitive dysfunction progresses more rapidly in patients with older PD onset, the frequency of MCI in young-onset patient s (around 50 years) being 5% compared to 25% in older ones (61 years plus; Santos-García et al., 2023). Women experience worse global cognitive decline during the prodromal phase of PD (Flores-Torres et al., 2023). REM sleep behavior disorder is a cognitive risk factor for individuals with newly diagnosed PD (Kenney et al., 2023; Nagy et al., 2023). Both diabetes/prediabetes and gut microbiome are also risk factors for PD-CI (Grant et al., 2023; Park et al., 2023).

Investigations which neuropsychiatric symptom profiles were associated with the risk of dementia in patients with PD-MCI showed that mood symptoms were associated with lower scores in verbal memory and executive domains, whereas hyperactivity symptoms were associated with lower scores in naming, visuospatial and verbal memory domains, and psychotic symptoms with lower scores in the visuospatial domain. This demonstrates that higher burden of neuropsychiatric symptoms is associated with dementia conversion (Lee et al., 2023).

In conclusion, recent genetic studies have shown that a number of well-described genes and PGS are risk factors for cognitive decline in PD, associated with impairment of general or specific cognitive phenotypes, although the identification of specific factors and their molecular interaction awaits further elucidation. Our understanding of the mechanisms behind these factors, as well as the interaction between gene and environment as determinants of disease phenotypes and the identification of modifying risk factors will be paramount, as this will contribute to potential intervention in this disorder.

## 5 Neuroimaging findings

MCI is common in PD but heterogeneous, with cognitive subtypes, but the underlying pathobiological mechanisms have not been fully understood. A combined analysis of voxel based morphometry (VBM) evaluating the regional changes combined with functional connectivity was used to explore the relevant morphological and functional changes and their association with CI in PD, but the findings are not always concordant (Hou and Shang, 2022).

Whole-brain analyses revealed increased global brain atrophy (Mak et al., 2017) or mild diffuse brain atrophy (Martin et al., 2009; Pereira et al., 2014). For major neuroimaging findings in PD-MCI see Supplementary Table 1.

### 5.1 Gray matter changes in PD-MCI

Structural gray matter (GM) changes associated with overall cognitive functions are mainly located in the frontal and limbic system, and are associated with subcortical atrophy. Atrophy of limbic lobes is associated with impaired memory, whereas frontal lobe atrophy is associated with executive dysfunction. Subtle brain structure changes in early PD CI and MCI stage can be detected via VBM (Gao et al., 2017). In PD-NC individuals, brain structures may be unchanged or show mild atrophy of the medial temporal cortex (Martin et al., 2009; Pereira et al., 2014), although, at baseline, PD-NC patients already presented cortical thinning in primary/premotor cortex, temporoparietal regions and striatum (Gorges et al., 2020).

In PD-SCD, GM atrophy was seen in the anterior cingulate and right parietal lobe (Hong et al., 2012; Chen et al., 2023). Compared to the HC group, PD-NC suffered from GM atrophy in prefrontal and limbic lobe and left temporal gyrus, while PD-MCI showed GM atrophy in frontal and limbic lobe, basal ganglia and cerebellum (Gao et al., 2017). Compared to PD-NC, the PD-MCI group exhibited GM atrophy in left middle temporal, inferior temporal gyrus and frontal lobe. Limbic atrophy was associated with impaired memory, frontal lobe atrophy with executive dysfunction. Subtle brain structure changes of the PD-early-CI and PD-MCI stages detected via VBM may be a neuroimaging marker for early diagnosis of PD-MCI (Gao et al., 2017). PD-MCI patients showed reduced gray matter volume (GMV) in the frontal cortex, extending to insula and cerebellum, and thinner cortex in the temporal lobe extending to the parietal cortex. GMV of the right middle frontal gyrus and cortical thickness of the right superior temporal gyrus were correlated with neurocognitive dysfunctions in PD-MCI (Li et al., 2022). PD-MCI patients showed decreased cortical thickness in the left superior temporal cortex and lingual, right insula and fusiform areas (Zhu et al., 2022), GMV reductions in left superior temporal, superior frontal lobe and left insula (Xu et al., 2016), in temporal and parietal cortex, putamen, amygdala, hippocampus and cerebellum (Melzer et al., 2012). PD-MCI patients exhibited GM reduction in the frontal and angular gyrus, precuneus, temporal lobe, and cerebellum. Early PD-MCI showed reduction of GM density in superior frontal gyrus and cerebellum that could be used as early marker fo PD-CI (Donzuso et al., 2021). Meta-analyses of PD-MCI cohorts reported GM atrophy in bilateral prefrontal cortex, insula, left angular gyrus, right supramarginal gyrus, midcingulate cortex, and right hippocampus (Mihaescu et al., 2019) or in left inferior and orbital frontal gyrus and left anterior insula (Zheng et al., 2019). Mild cortical atrophy affected orbitofrontal cortex, left superior parietal lobule and more widespread limbic and fronto-parietal cortex (Kunst et al., 2019). Cortical thinning of temporal, parietal and occipital cortices was correlated with reduced visuospatial/perceptual functions, widespread non-specific anterior-posterior cortical thinning with reduced EF (Garcia-Diaz et al., 2018).

Several structural MRI studies focused on assessing subcortical GM structures. PD-MCI compared with PD-NC showed reduced volumes of right caudate nucleus, thalamus, and right hippocampus-amygdala transition (Foo et al., 2016). PD-MCI was associated with atrophies in the amygdala, CA1 subregion and hippocampal subiculum (Zhang L. et al., 2023). Independent of disease duration, patients with PD-MCI showed significantly smaller hippocampal volumes than PD-NC, which were associated with worse performance in EF, memory, language and spatial working memory tests (Becker et al., 2021). PD-MCI showed microstructural degeneration in bilateral cingulate and paracingulate gyri, supplementary motor area, right paracentral lobule and precuneus, while PD-MCI showed lower neurite orientation dispersion index (ODI) in widespread regions covering bilateral frontal, parietal and right temporal areas. Reduced ODI in right frontal area and bilateral caudate nucleus was associated with MoCA scores and memory performance. This indicated that cortical microstructural alterations may precede macrostructural lesions (Bai et al., 2022). In order to detect the earliest signs of PD-MCI, measures that are sensitive to earlier pathological events are needed. These are likely to be techniques such as diffusion tensor imaging that detect axonal and synaptic changes (Hattori et al., 2012) which occur at earlier stages in PD-MCI.

PD-MCI patients showed baseline thalamus atrophy and progressive atrophy in caudate nucleus, presubiculum and hippocampus, associated with executive and memory dysfunctions (Foo et al., 2017). Fronto-striatal metrics were associated with faster attention/execution decline, whereas lower baseline hippocampal volume correlated with faster global cognitive decline, particularly memory tasks (Brown et al., 2023). Early PD-MCI showed atrophy of the right entorhinal cortex (Jia et al., 2019), subcortical limbic structures (Hanganu et al., 2014), and atrophy of hippocampal subfields, while reduction of left para- and presubiculum predicted conversion from PD-NC to MCI (Becker et al., 2021). Hippocampal volumes differed significantly between PD-NC and MCI (Yazdan Panah et al., 2023), while atrophy of subcortical structures (thalamus, putamen, caudate nucleus and amygdala) showed an overlap of PD-MCI and PD-NC (Sivaranjini and Sujatha, 2021). In addition, the PD-MCI group, compared with the PD-NC ones exhibited atrophy of entorhinal cortex (Goldman et al., 2012b; Jia et al., 2019), amygdala (Schulz et al., 2018), nucleus accumbens (Mak et al., 2015), and nucleus basalis of Meynert (NBM; Schulz et al., 2018).

Few neuroimaging studies have considered cognitive heterogeneity within PD-MCI, making it difficult to draw robust conclusions regarding the anatomical-functional bases of specific subtypes. Studies comparing PD-MCI with HCs and/or PD-NC found brain modifications in parietal, occipital and temporal regions in patients with aMCI and, to a lesser extent, naMCI; with executive MCI in frontal and striatal regions, and with non-executive MCI in posterior cortical regions (Devignes et al., 2022). In the PD-MCI group, NBM volume was positively correlated with cortical thickness in bilateral cingulate, parietal, frontal, and left angular regions. NBM atrophy was more correlated with cortical thickness of medial orbito-frontal lobe, right posterior and anterior cingulate and precuneus in PD-NC vs. HCs, and the right precuneus and posterior cingulate in PD-MCI. The stronger correlation between NBM and cortical thinning in PD-MCI suggests that NBM volume loss may play an important role in PD-CI (Rong et al., 2021). PD-MCI was associated with mild midbrain atrophy and cortical thinning in lateral orbitofrontal regions (Cicero et al., 2022a). Comparison of the volumes of amygdala nuclei showed significant differences between PD-NC, PD-MCI, and HCs: the left cortico-amygdaloid transition area and the left superficial cortex-like region volumes classified cognitively normal and cognitively impaired PD with up to 90% accuracy and, thus, discriminated CI in PD (Ay et al., 2023).

Longitudinal MR studies described the progressive patterns of GM changes in patients with PD-MCI with increased global higher rate of cortical thinning in the frontal/supplementary motor area, temporo-parietal, occipital cortices and decrease in the volume of amygdala and nucleus accumbens relative to both PD-NC and HCs (Hanganu et al., 2014; Mak et al., 2017). A 4-year follow-up study reported that PD-MCI patients showed significantly greater progression of cortical thinning in the posterior cortical regions related to visuospatial and visuoperceptual changes (Garcia-Diaz et al., 2018). Another 4-year follow-up study showed different vulnerabilities of hippocampal subfields, with decline in anterior and posterior hippocampal segments engaged in memory dysfunctions (Uribe et al., 2018).

### 5.2 White matter changes in PD-MCI

White matter (WM) lesions often precede GM atrophy and were observed in early PD with intact GM (Agosta et al., 2014; Duncan et al., 2016; Rektor et al., 2018). PD-MCI patients had significant WM alterations compared with PD-NC (Liao et al., 2023), particularly periventricular and deep WM hyperintensity (WMH) load being significantly higher (Zhao et al., 2023). Periventricular WMHs were associated with PD-MCI, whereas total brain WMH burden was not. WMH volume was associated with impairment of global cognition, EF and language (Scamarcia et al., 2022), heavier periventricular WMH burden with worse EF and visuospatial function (Huang et al., 2020). However, WM volume reduction was not a consistent finding in PD-MCI compared with HCs (Hanning et al., 2019; Butt et al., 2021), and it often was not correlated with cognitive function in early PD (Dalaker et al., 2009), suggesting that WM damage may not be relevant for some phenotypes of PD-MCI (Hall and Lewis, 2019). On the other hand, microstructural changes in WM tracts were present even in early phases of PD (Sarasso et al., 2021). Total and periventricular WMH burden at baseline predicted decline in global cognition, total WMH burden that of EF, occipital WMH that in visuomotor attention and visuospatial memory, while WML at baseline did not predict motor decline (Carvalho de Abreu et al., 2023). Recent studies also stated that microangiopathic WML did not have a relevant impact on neurocognitive performance in PD, which was related rather to neuronal dysfunction (Schröter et al., 2023), while WM structural connectivity was considered an early and sensitive indicator of future MCI conversion in *de novo* PD patients (Huang X. et al., 2023).

PD-MCI showed distinct degeneration pattern in the association fibers; fractional anisotropy (FA) in the right fronto-occipital fascicle was positively correlated with EF, as was the mean diffusivity (MD) of the left superior longitudinal fascicle, demonstrating regional tract-specific microstructural changes of association fibers (Yu et al., 2023). Voxel-based FA values in PD-MCI decreased in bilateral frontal and temporal lobes, bilateral subthalamic nucleus, corpus callosum and cingulate gyrus. They also decreased in bilateral corticospinal tract, anterior and posterior cingulum, corpus callosum, bilateral fronto-occipital and bilateral parieto-occipital tracts, indicating abnormalities in multiple brain areas and WM tracts (Pu et al., 2020). Widespread WM damage in terms of reduced FA and increased MD were reported over 1–2 years in *de novo* PD with MCI (Minett et al., 2018; Pozorski et al., 2018; Taylor et al., 2018; Rau et al., 2019). PD-MCl relative to PD-NC patients showed reduced FA in WM frontal regions over 1.5 years and reduced FA at baseline predicted decline of EF over time (Minett et al., 2018). Baseline and longitudinal reduced WM volume correlated with cognitive performance and predicted conversion to PD-MCI (Wen et al., 2015). Reduction of the corpus callosum and major association tracts was seen in PD-MCI but not in PD-NC (Agosta et al., 2014; Chen et al., 2016). Thinning of the corpus callosum and cingulum correlated with reduced thickness of the left orbitofrontal cortex (Owens-Walton et al., 2022). Early PD-MCI was associated with poorer olfactory function and disordered WM integrity in anterior olfactory structures (Stewart et al., 2023).

PD-MCI patients had significantly lower neurite density and orientation dispersion index in WM clusters in the prefrontal region, the cingulum bundle and thalamo-frontal clusters, representing local WM abnormalities (Zhang C. et al., 2023). The body of the corpus callosum and superior corona radiata were significantly reduced; the cingulum, superior longitudinal fasciculi and thalamocortical circuit exhibited fiber bundle changes (Liao et al., 2023). More comprehensive information on WM changes can be obtained by combining intra- and intervoxel diffusion tensor imaging indices, showing that the local diffusion homogeneity of the brainstem and hippocampus was an important feature in PD-MCI (Chen et al., 2023). In addition to WMHs, other metabolic features were important for CI, in particular, homocystein levels that were negatively related to visuospatial/executive functions, while WMHs correlated with global cognition (Kobak Tur and Ari, 2023). Periventricular WMHs combined with plasma homocysteine levels were able to predict PD-MCI (Zhang Z. et al., 2023).

In conclusion, GM changes in PD-MCI involve not only prefrontal, orbitofrontal, temporal, and limbic (hippocampal) regions but also extensive subcortical structures including thalamus, striatum, amygdala and cholinergic forebrain structures. WM lesions affect diffuse and periventricular areas, corpus callosum and widespread association tracts, microstructural lesions representing earliest lesions. Both GM and WM changes differentiate PD-MCI from PD-NCI and HCs not only by the severity of these lesions but also their extension, which may be important for the progress of cognitive dysfunctions in early PD.

## 6 Neuroimaging changes in PD-MCI converters

PD patients with normal cognition at baseline (PD-NC) who develop MCI during follow-up are classified as “PD-MCI converters,” while those PD-MCI patients who became asymptomatic during the follow-up are referred to as “non-converters.” PD-MCI converters have a reduced GM volume of temporal regions and the amygdala-hippocampus network at baseline, associated with early right temporal atrophy, progressive frontal lobe atrophy and WM volume reduction (Wen et al., 2015; Zhou et al., 2020; Chen et al., 2021). Others described GM thinning in anterior cingulate, temporal, parietal and occipital cortices, progressive atrophy of the caudate nucleus, thalamus, nucleus acccumbens and CA2/3 hippocampal subregion (Foo et al., 2017; Filippi et al., 2020; Gorges et al., 2020).

VBM analysis of PD-MCI converters showed lower GM density in the left prefrontal areas, left insular cortex, and bilateral caudate nucleus, and smaller substantia innominata volume compared with non-converters (Lee et al., 2014). Subcortical shape analysis revealed smaller volumes in the bilateral thalamus, right caudate nucleus and right hippocampus, thalamic local shape volume being associated with semantic fluency and attentional composite score (Chung et al., 2017). However, multivariable logistic regression revealed no significant differences between converters and non-converters regarding the extent of WMH or within cholinergic pathways (Hanning et al., 2019), whereas others showed that PD-MCI converters had larger WMH volume and higher hyperintensitive score compared with non-converters (Sunwoo et al., 2014).

PD-NC and PD-MCI patients who progress to PDD at follow-up are classified as “PDD converters,” who, at baseline have shown lower GMV in prefrontal and left insular cortex extending to posterior cortical areas, bilateral striatum, and the amygdala/hippocampus structural covariance network (Chung et al., 2019; Filippi et al., 2020; Zhu et al., 2022). These and other changes in PDD converters have been reviewed recently (Jellinger, 2024) and are not the subject of the present paper.

## 7 Brain network connectivities in PD-MCI

Structural brain atrophy may not be associated with any cognitive domain, with the exception of visuospatial measures in primary sensory and motor cortices, whereas functional connectivity (FC) is usually associated with attention, EF, language, learning, memory, visuospatial, and global cognition in multiple brain areas (Wylie et al., 2023).

The high-level cognitive processes are supported by intrinsic brain networks, as evidenced by resting-state functional MRI (rs-fMRI), and brain connectivity markers can predict MCI (Lin et al., 2021).

**Figure 1:**
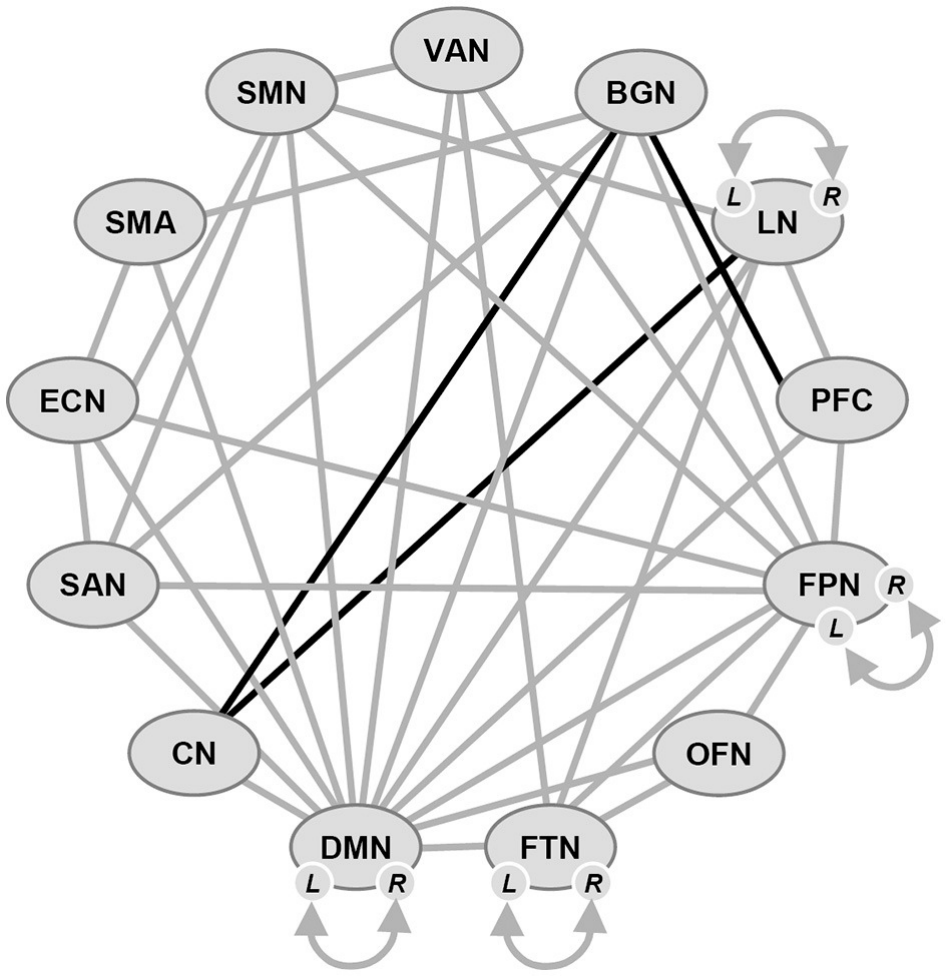
Schematic overview of functional connectivity in some major networks in Parkinson's disease with mild cognitive impairment (PD-MCI). Gray lines: hypocommunication, black lines: hypercommunication. L, R: left, right hemisphere. Network abbreviations: VAN, ventral attention; BGN, basal ganglia; LN, limbic; PFC, prefrontal cortex; FPN, frontoparietal; OFN, orbitofrontal; FTN, frontotemporal; DMN, default mode; CN, cerebellar; SAN, salience; ECN, executive network; SMA, supplementary motor area; SMN, sensorimotor; N always meaning network.

Eight major hierarchically organized resting state networks (RSN) serving higher-level cognitive functions have been identified, namely the visual (VN), sensori-motor (SMN), dorsal attention (DAN), ventral attention (VAN), limbic (LN), fronto-parietal (FPN), salience (SAN), and default mode (DMN) networks (Yeo et al., 2011; Seitzman et al., 2019), plus the executive (EN) and the subcortical (striatal; SCN) networks. Four of these RSNs (SMN, DAN, VAN, and FPN) have specifically been implicated in the pathological dysfunctions present in PD-MCI patients (see Figure 1).

The DMN, which is believed to serve an important role in various cognitive functions, includes the medial parietal, bilateral inferior-lateral parietal and ventromedial-frontal cortex (Smith et al., 2009). In PD, changes in its connectivity have been reported (Tessitore et al., 2012; Disbrow et al., 2014; Yao et al., 2014). Alterations in regions related to the DMN in PD (Tahmasian et al., 2017) support findings which indicate its involvement in cognitive decline in PD (Wolters et al., 2019). The FPN is a critical component in EFs and working memory (Petersen and Posner, 2012) and related to dopamine depletion in PD (Lang et al., 2019), while attentional orienting is driven by both the VAN and DAN (Peraza et al., 2017; Bezdicek et al., 2018), which has been associated with PD-MCI (Lebedev et al., 2014; Caminiti et al., 2015). They are associated with the cholinergic neurons in the basal forebrain, and the cholinergic neurotransmitter loss has been linked to attention deficits in this system (Ballinger et al., 2016). The SMN serves primary motor functions, but it has also been associated to both impaired sensory integration for motor function (Chen et al., 2021) and the fronto-executive dysfunction cognitive profile in PD, mainly impacting the FPN. Thus, it plays an important role in verbal short-term memory, coordinating with the fronto-temporal areas (Buchsbaum and D'Esposito, 2019). Furthermore, the SMN cognitive role arises from its disruption in association with CI in PD (Agosta et al., 2010; Chen et al., 2020). The SAN, thought to be involved in maintaining vigilance and arousal, is composed of inferior anterior insula and anterior cingulate cortex (Seitzman et al., 2019). It is a key neuronal substrate of CI in PD (Yeager et al., 2023).

Three networks, the DMN, FPN, and SAN have been linked to EF, with the SAN playing a role in modulating DMN and FPN activity (Seeley et al., 2007; Sridharan et al., 2008; Bressler and Menon, 2010). The SAN integrating responses to salient stimuli (Menon and Uddin, 2010), its control over the DMN and FPN is dysregulated in PD and aging (Putcha et al., 2016; Chand et al., 2017). The basal ganglia network (BGN) is also of significance to PD, because dopaminergic deficits in the basal ganglia can profoundly alter functional brain networks (Obeso et al., 2000; Shafiei et al., 2019; Shima et al., 2023). The relationship of SAN dysfunction to other brain networks and CI are unclear (Badea et al., 2017), as is BGN connectivity with essential cortical networks (DMN, SAN, and FPN; Yeager et al., 2023).

New techniques for quantification of structural changes across the entire network are emerging that are likely to show sensitivity for changes in PD-MCI (Nigro et al., 2016). Compared to HCs, PD-MCI patients displayed reduced between-network connectivity of large functional networks related to cognition, which increased with time and MCI status, reflecting compensatory efforts (Klobušiaková et al., 2019). Brain connectivity analyses revealed network connectivity changes in both PD-NC and PD-MCI groups compared to HCs. It should be emphasized that FC changes within the most relevant neurocognitive networks were already detectable in early drug-naive PD patients even in the absence of compensatory mechanisms (De Micco et al., 2023). Compared to HCs, PD-MCI patients had a large BGN and FPN with decreased FA in the right hemisphere and a subnetwork with increased MD involving similar regions bilaterally. The PD-MCI group showed a significant reduction in FC between the DMN and the precentral gyrus, middle temporal cortex insula, middle frontal gyrus and anterior inferior parietal lobule and a significantly decreased FC in the middle frontal and temporal gyri. Furthermore, PD-MCI patients had a network with decreased FA, including basal ganglia and fronto-temporo-parietal regions bilaterally (Galantucci et al., 2017).

Disruption of structural connections between brain areas forming a network contributed to differentiate between PD with and without MCI (Galantucci et al., 2017). The PD-MCI group had significantly decreased FC within the DMN, mainly between the hippocampal formation and inferior frontal gyrus, between the posterior cingulate gyrus and posterior inferior parietal lobule, and between anterior temporal lobule and inferior frontal gyrus. The decreased FC between anterior temporal lobe and middle temporal gyrus was positively correlated with attention/working performance, and the reduced FC between hippocampus and inferior frontal gyrus with memory function. These findings indicated that an altered DMN connectivity characterizes PD-MCI (Hou et al., 2016). Loss of GM was observed in the DMN (bilateral precuneus) without a corresponding disruption of functional properties, whereas these appeared in the SAN. There was a correlation between visuospatial scores and right supramarginal gyrus node degree. These findings highlight the loss of FC and topology without structural damage in the SAN regions in PD-MCI. Functional disruption in the absence of GM atrophy suggests that the SAN is a key vulnerable system at the onset of PD-MCI (Aracil-Bolaños et al., 2019). Comparison between PD-NC and HCs further showed a selective decline in interconnectedness between bilateral lentiform nuclei, and between PD-NC and MCI in the bilateral superior parietal lobules and precuneus. The connectivity changes were localized in the hubs of the posterior attention network. Aligned with the predominant attention deficit, this seemed to be a hallmark of PD-MCI (Bezdicek et al., 2018).

Both PD-NC and PD-MCI patients compared with HCs showed decreased DMN connectivity, while only PD-MCI ones showed decreased FC of bilateral prefrontal cortex within the FPN. In both early PD-NC and MCI PD, the topological organization of essential GM networks—SMN, DMN, SAN, and FPN—were disrupted, as well as the connections between DMN and cerebellar network, which were greater and more extended in PD-MCI (Suo et al., 2021): a significant difference between PD-MCI vs. PD-NC and HCs was in the FC-trait comprising SMN, DAN, ventral attention VAN and FPN networks, which was associated with working memory, and memory. Intra-network changes were found in resting state networks related to attention, EF and motor control. Interaction between the SMN, and DAN, VAN and FPN networks reflects the intertwined decline in motor and cognitive abilities in PD-MCI. This suggests that the memory impairment observed in PD-MCI is associated with reduced FC within the SMN and between SMN and attention networks (Delgado-Alvarado et al., 2023).

The decreased prefrontal cortex connectivity correlated with cognitive parameters but not with other clinical parameters, suggesting that altered DMN connectivity characterizes PD patients regardless of cognitive status, whereas functional disconnection could be associated with PD-MCI even in the absence of detectable structural changes (Amboni et al., 2015). Decreased cortical thickness in left superior temporal and fusiform, right insula and fusiform areas in PD-MCI resulted in decreased FC between these affected regions contributing to cognitive decline (Zhu et al., 2022). PD-MCI showed 17 WM circuits with reduced connectivity compared to HCs, mainly involving temporal/occipital regions. Reduced structural connectivity in fronto-striatal and cortico-cortical connections was associated with PD-MCI (Inguanzo et al., 2021). It further induced interrupted, slow evolution of subnetworks, predominantly linking temporal-parietal-occipital lobes, in functional brain network dynamics in early PD, and the dynamic expression characteristics of these subnetworks also reflected the degree of CI in early PD (Chu et al., 2023).

FC analysis performed with cholinergic forebrain nuclei, for the right NBM/Ch4 exhibitd lower FC in the right middle cingulate and paracingulate gyri, middle frontal, left inferior parietal and superior frontal gyri compared to HCs, and significantly lower FC in left putamen, middle frontal gyrus, cingulate and paracingulate gyri compared to PD-NC. Increased FC was seen in right calcarine fissure, surrounding cortex and cerebellum. Altered FC involved the cortical regions of NBM/Ch4. For the nucleus of the diagonal band CH1-3, FC values in the right middle cingulate and paracingulate gyri were reduced. These changes indicate functional alterations within the cholinergic system (Zhang P. et al., 2023).

PD-MCI was associated with reduced FC of the mediodorsal thalamus and para- and posterior cingulate cortex, while its connection to posterior cingulate cortex was increased (Owens-Walton et al., 2021): further decreased FC between the striatal networks was partly due to atrophy within the SAN, as were the connections between right caudate nucleus an anterior circulate cortex, precuneus and left supramarginal gyrus, while FC to left hippocampus and right cerebellar hemisphere was increased (Lang et al., 2020). PD-MCI further demonstrated poor olfactory functioning and abnormalities detected by diffusion tensor imaging in the anterior olfactory structures, relative to PD-NC individuals. Olfactory defect and microstructural changes in the anterior olfactory structure may be an additional biomarker of PD-MCI (Stewart et al., 2023).

In conclusion, both early and PD-MCI are characterized by reduced FC in the DMN and SAN, the inferior frontal and anterior temporal cortex, the left and right FPN bilateral superior medial frontal cortices, with increased FC in SAN and FPN, but breakdown of the connection between mediodorsal thalamus and the cingulate cortex, as well as the striatal network and its cortical connections, indicating a dysfunction of multiple attentional cognitive control and other essential networks.

## 8 Brain metabolic and other PET studies

18F-Fluorodeoxyglucose PET (18F-FDG PET) studies in PD-MCI patients revealed reduced metabolism in the frontal lobe and to a lesser degree in parietal and occipital areas compared to HCs. Mini-mental state examination (MMSE) correlated positively with metabolism in several lobes, EF with metabolism in the parieto-occipito-temporal junction and frontal lobe, visuospatial function with parieto-occipital and language with frontal metabolism (Garcia-Garcia et al., 2012). In PD-MCI patients hypometabolism exceeded GM atrophy in angular gyrus, orbital, anterior frontal and occipital lobe, indicating a gradient of severity of cortical changes associated with the development of CI (González-Redondo et al., 2014). Other PET studies in PD-MCI showed reduced metabolism in posterior cortical regions, particularly in parietal and occipital cortex, which were not affected in PD-NC (Homenko et al., 2017; Schrag et al., 2017). In comparison to both PD-NC and HCs, PD patients with MCI exhibited hypoperfusion in the parietal memory network and decreased precuneus FC in the right striatum, which was positively associated with memory dysfunction (Jia et al., 2019). PD-MCI patients had reduced regional cerebral blood flow (CBF) in the left precentral and middle cingulate gyrus, right middle frontal gyrus and bilateral putamen compared to the aMCI group. Correlations to EF were found in the PD-MCI group, and correlations to memory peformance in the aMCI group (Shang et al., 2021). After 2-year follow-up, PD-MCI patients showed significantly reduced CBF in multiple prefrontal regions. More importantly, converters to PD-MCI showed more significant CBF reduction in the left lateral orbitofrontal cortex than non-converters. These findings suggest that longitudinal CBF reduction in the prefrontal cortex might impact cognitive function (especially EF) at early stages of PD (Wang J. et al., 2022). Brain metabolism related to MCI and phenoconversion in patients with isolated REM sleep behavior disorder (iRBD), a risk facor for subsequent PD, was reduced in the inferior parietal lobule, lateral and medial occipital and middle and inferior temporal cortex bilaterally compared with HCs and the iRBD-NCI group (Yoon et al., 2022). A cognitively stable PD-NC group had frontal predominant hypometabolism, while PDD converters showed parieto-occipital hypometabolism at baseline regardless of whether a patient's initial cognitive status was PD-NC or PD-MCI (Baba et al., 2017). In PD-MCI, impaired self-awareness of cognitive deficits was related to decreased metabolism in the right superior temporal lobe, insula and midcingulate cortex, but not to cortical thickness in these regions (Maier et al., 2023). Decreased metabolism in left frontal and posterior cortex was related with executive and memory dysfunction, occipital hypometabolism with visuospatial dysfunction, suggesting that its level in a specific brain region may indirectly reflect the relevant cognitive dysfunction (Zhihui et al., 2023). MCI subgroups showed overlapped impaired regions. They had reduced cerebral blood flow in the bilateral putamen, left precentral, and middle cingulate gyrus, and right middle frontal gyrus compared to aMCI. CBF in the left precentral, left middle cingulate and right middle frontal gyrus was significantly altered (Shang et al., 2021).

PD-MCI in the posterior cingulate cortex exhibited reduced N-acetyl aspartate (NAA), total NAA, choline, glutathione, glutamate + glutamine, and total creatine (tCr) levels but both elevated myo-inositol (Ins) and Ins/tCr ratio, but reduced NAA/Ins ratio. ROC curve analysis revealed that tCr concentration could differentiate PD-MCI from controls, while PD-NC individuals with low NAA and tCr may be at risk of preclinical PD-MCI (Huang M. et al., 2023).

Earlier 18F-florbetapir PET studies in PD-NC subjects showed extremely low β-amyloid (Aβ) positivity (Mashima et al., 2017), although regional cortical Aβ deposition in the frontal cortex, precuneus and anterior or posterior cingulate gyrus inversely correlated with naming and verbal memory performance, respectively (Akhtar et al., 2017), while others found no such association (Ko et al., 2017; Melzer et al., 2019). More recent studies detected higher Aβ deposition in prefrontal and temporal cortex in PD-MCI (Ghadery et al., 2020). Aβ-positive aMCI cases had lower dopamine activities in the left striatum, suggesting changes related to AD pathology (Oh et al., 2021); others found significant correlation between plasma Aβ-42 level and FA in multiple regions of the left brain, as well as between plasma tau levels and the average thickness of the posterior cingulate gyrus (Huang C. C. et al., 2023). The finding that Aβ deposition was located mainly within the DMN suggested disturbances in Aβ topological organization characterized by abnormal network integration/segregation and the spreading pattern of Aβ between brain modules in these patients (Kim et al., 2019). Other studies suggested that regional Aβ deposition alone has a moderate effect on predicting future cognitive decline in PD and that the patchwork effect of Aβ deposition on cognitive ability may separate CI from cognitive sparing in PD (Mihaescu et al., 2022). Cerebral Aβ worsened executive functions but not the global cognitive abilities and was not associated with middle-temporal cortex atrophy, suggesting that Aβ alone may not be the main pathogenetic determinant of cognitive deterioration in PD-MCI, but would rather aggravate deficits in domains vulnerable to PD primary pathology (Garon et al., 2022). 18F-flortaucipir PET, detecting tau deposition in brain tissue, showed no or minimal tau binding in PD-NC and minimal in PD-MCI as compared to low/moderate in PD (Bohnen et al., 2017).

## 9 Neuropathological findings in PD-MCI

While the heterogeneous pathology of PDD and MCI in other dementing disorders is well-documented (Markesbery, 2010; Halliday et al., 2014; Jellinger, 2022, 2024), little is known about the specific neuropathology of PD-MCI, since there are only few detailed studies in small cohorts. Among 698 autopsy-proven cases of PD, 16 met the clinical criteria of PD-MCI (Adler and Beach, 2010; Jellinger, 2013). They included 7 naMCI cases (mean age at death 80 years, mean duration of disease 9.7 years), 50% brainstem predominant, 31% brainstem-limbic and 19% neonatal LB stages, Braak neuritic stages 0–4 (mean 2.1), two with few neuritic plaques and 4 with Aβ plaques, 8 aMCI cases (mean age 80 years, mean disease duration 15.1 years), brainstem predominant 4, brainstem-limbic 3 and neocortical stage one. Braak NFT stage I-IV (mean 2.7), 4 each with moderate neuritic plaques and with many Aβ plaques. One single aMCI MuD case (aged 75, 11 years disease duration), brainstem type, neuritic Braak stage II, with few neuritic and many Aβ plaques. Co-pathologies included mild cerebral amyloid angiopathy (CAA) in the aMCI MuD case, mild lacunar state in basal ganglia in four cases and old brain infarct in one. Braak NFT stage in aMCI cases was higher than in naMCI ones, mild to moderate neuritic plaques were present in 43% (no differences among MCI subtypes), mild CAA in 11%, lacunar state in 25% (similar in all MCI subtypes), and old cerebral infarcts in 12% (one aMCI and naMCI case each).

Another autopsy study of 159 PD cases included 25 MCI ones (56% aMCI and 44% naMCI) with no significant differences in age, gender and disease duration. All naMCI cases were brainstem-limbic LB stage 3, in the aMCI group 22% were LB neocortical stage 4. Concomitant tau pathology was present in nine cases, both aging-related tau astrogliopathy (ARTAG) and argyrophilic grain disease (a 4R-tauopathy) were present in five cases, while two aMCI cases met the pathological criteria of progressive supranuclear palsy. No differences were found in neuritic plaque stage, total Aβ and CAA score, WM rarefication, cerebral infarct volume and APOE carrier frequency. Slight differences between MCI subtypes were higher Braak NFT stage in aMCI cases and mild increase in LB pathology in naMCI-PD cases (Knox et al., 2020).

In conclusion, the few available neuropathological data gave a heterogeneous picture of PD-MCI with a combination of various types and degrees of relevant changes and co-pathologies that were comparable with MCI in other diseases (Markesbery, 2010; Takao, 2012). Therefore, the morphological basis of the different subtypes of PD-MCI and the impact of frequent co-pathologies warrant further elucidation.

## 10 Biomarkers for PD-MCI

Biomarkers for CI in PD could aid in both diagnostic and prognostic evaluation and in the development of new cognitive enhancing treatments. Promising biomarkers for MCI in the preclinical stage of PD are: (1) description of the cognitive profile in several subdomains applying the MDS-TF (level II) criteria; (2) 123I-FP-CIT SPECT examining degeneration of the dopaminergic nigrostriatal pathway and/or DAT transporter and/or 18F-FDOPA PET examination; (3) FDG-PET investigating brain metabolic signature; (4) EEG markers or calculating delta/theta rhythm frequency, analyzing peak frequency, etc.; (5) assessment of plasma and/or CSF levels of αSyn and/or using of seeding amplification assays of αSyn in CSF and/or plasma (quaking induced conversion assay); (6) assessment of CSF and/or plasma Aβ-40 and−42, total (t)-tau and phosphorylated (p) tau 181; (7) measurement of CSF and/or plasma neurofilament light-chain (NfL) and glial fibrillary acidic protein (GFAP) levels; (8) assessment of serum glial cell line-derived neurotrophic factor (GDNF); (9) assessment of levels of CD8+, TNF-α, IL-6, Treg, and other inflammatory-related markers; (10) assessment of αSyn, p-tau and Aβ-42 in saliva; (11) MRI findings detecting local brain atrophy using VBM and other technologies, WMH burden and functional brain network changes; (12) assessment of cortical acetylcholinesterase activity by both PET and MRI imaging; (13) combined MRI imaging and EEG examination detecting widespread structural and EEG abnormalities; (14) detection of olfactory deficits and microstructural changes in the anterior olfactory structure. Combinations of biomarkers could be valuable for the individualized diagnosis of MCI as indicator for cognitive changes in earliest or prodromal stages of PD.

### 10.1 EEG biomarkers

PD-MCI is characterized by widespread functional, structural and EEG abnormalities, and electrocortical abnormalities (decreased and increased network activities) may represent the instrumental counterpart of early cognitive decline in PD (Mostile et al., 2019). Changes in brain activities were limited to distinct cognitive domains, especially reduced beta power in the frontal region could serve as an electrophysiological marker for CI in non-demented PD (He et al., 2017). Both PD-NC and MCI patients had diminished alpha and theta phase oscillations compared to HCs, but electrophysiological abnormalities were more pronounced In PD-MCI over frontal, parietal, and temporal locations in almost all frequency bands, accompanied by bilateral thalamus, putamen and hippocampus atrophy (Hünerli-Gündüz et al., 2023). Both PD-NC and MCI had decreased alpha and delta frequencies over frontal, parietal and temporal regions, the most significant decreases were shown in the left frontal-right occipital and left occipital-right frontal areas (Zawislak-Fornagiel et al., 2023). PD-MCI patients with a frontostriatal subtype displayed higher powers in the delta and theta bands, lower powers in the beta2 band and lower FC in the beta2 band compared to PD-NC and -MCI with posterior cortical type, which were mainly located in the frontal, limbic, and parietal regions. This showed the promising potential of EEG to discriminate between PD-MCI subtypes (Betrouni et al., 2022). Other differences were a decreased network involving alpha activity over the occipital lobe, increased network involving beta activity, an increased delta and theta activity over the frontal lobe, associated with reduction involving delta and theta activity in the parietal lobe. Quantitative EEG analysis showed a significant decrease of alpha power spectral density (PSD) over the occipital regions and increased theta PSD over the left temporal region (Mostile et al., 2019). The temporal correlation coefficients of PD-MCI patients were lower in the theta and delta bands than those of PD-NCI cases, and the delta and alpha bands were lower in PD-MCI (Yi et al., 2022). Compared to controls, they showed significantly lower alpha2 power and alpha2/alpha1 ratio, and significant higher delta and lower beta power and alpha/delta; MoCA score correlated inversely with delta power and directly with alpha2 and beta power, as well as with alpha2/alpha1 and alpha/delta ratio (Polverino et al., 2022). Decreased alpha, beta and delta activities were found in both the dorsolateral prefrontal cortex and caudate nucleus correlating with cognitive dysfunction (Paulo et al., 2023). The degree of CI was related to a decrease in the coherence in the alpha ranges in the left frontal-left parietal region rather than the right frontal-right parietal region (Mano et al., 2022). A significant joint effect of interventions on EF and a trend on attention was found for alpha power and a negative between attention and delta power, supporting the role of delta and alpha power at frontal regions as biomarker for cognitive decline (Trenado et al., 2023). PD patients showed slower alpha bands in frontal lobe areas and reduced 18F-FDOPA PET/CT uptake in putamen and caudate nuclei, along with a decreased putamen-to-caudate ratio, and longer performance times evident in nearly all EF test parameters. Thus, EEG wave slowing in the frontal lobes was correlated with striatal dopaminergic deficiency and impaired EF in mild PD, as a possible biomarker of PD-related EF (Lorek et al., 2023). Comparing recordings between cognitively healthy individuals and those with MCI revealed a smaller decrease in beta power in the caudate and dorsolateral prefrontal cortex during memory recording, suggesting that dysfunction of the cognitive cortico-striato-thalamo-cortical circuits could contribute to cognitive symptoms in PD (Hnazaee and Litvak, 2023; Paulo et al., 2023). PD-MCI patients further showed left and right posterior middle frontal gyrus-based changes in the frequency bands (Cai et al., 2021). Evoked mid-frontal theta/delta rhythms directly related to cognition in PD, suggesting that cognitive dysfunction results from decreased theta/delta activity (Singh et al., 2023). Furthermore, frequency-dependent microstate frequencies showed significant differences in the 1–11.5 Hz spectrum between PD-MCI and HCs. In this characteristic frequency, PD-MCI patients exhibited a pattern of global microstate disorder, which may enhance our understanding of cognitively related brain dynamics (Liu C. et al., 2023).

Magnetoencephalography showed higher power in lower frequency bands (theta and delta) associated with memory, language attention and global cognition. Widespread group differences were found in the beta band, with significant changes between normal condition and MCI groups. Bilateral frontal and left hemispheric regions were affected in the other frequencies as cognitive decline became more pronounced. This suggested that PD-MCI and PDD are qualitatively distinct cognitive phenotypes and most neurophysiological changes may occur during that time of transition (Simon et al., 2022). Modern resting state EEG measuring synchronous changes are future biomarkers for monitoring cognition in PD (Anjum et al., 2024).

In conclusion, EEG in PD-MCI displays multiple electrophysiological abnormalities predominantly in (pre)frontal, limbic and parietal regions or cortico-subcortical networks but also in posterior areas. Changes of alpha and theta power in frontal regions or corticostriatal beta oscillation changes could be EEG biomarkers for cognitive decline in PD. Modern resting state EEG measures of synchronous changes may be future biomarkers for monitoring cognition in PD.

### 10.2 Fluid biomarkers

The association between reduced CSF αSyn concentrations and concentration and cognition suggests that αSyn pathology contributes to early CI in PD, in particular to executive-attentional dysfunction (Skogseth et al., 2015). PD-MCI patients had lower CSF levels of total αSyn, Aβ-38,−49 and−42, t-tau and p-tau, compared to PD-NC. CSF Aβ-42 level in PD-MCI was lower than that in PD-NC, while t-tau and p-tau were elevated (Hu et al., 2017). Increased plasma levels of αSyn and p-tau 181 discriminated *de novo* PD patients from HCs and were suggested promising biomarkers for *de novo* PD patients (Ren et al., 2022). CSF Aβ-42 level did not correlate with reduction of hippocampal volumes, which in MCI were significantly smaller than in PD-NC (Becker et al., 2021), whereas significant correlation was found between plasma Aβ-42 and FA in the left middle temporal, angular and middle occipital gyri, as well as between plasma t-tau level and the thickness of the right cingulate gyrus (Huang C. C. et al., 2023). Plasma αSyn was lower in PD compared to controls and in PD-MCI compared to PD and controls, while Aβ-42 did not differ between groups. p-Tau 181 was higher in PD-MCI compared to PD-NC and controls, while t-tau did not differ between groups. The PD-MCI group had significantly higher plasma p-tau 181 levels and p-tau-181/Aβ-42 ratio and lower Aβ-42/Aβ-40 ratio compared to PD-NC. The combination of clinical features, plasma biomarkers, right occipital pole thickness and increased FC between left posterior cingulate cortex and left parahippocampal gyrus had the highest diagnostic accuracy for PD-MCI (*p* = 0.001; Wang Y. et al., 2022).

Tyrosine-phosphorylated insulin receptor substrate 1 was lower in PD-MCI than in PD-NC and controls, the ratio of αSyn to p-tau 181 was lower in PD-MCI than in PD-NC and HCs; the ratio of insulin receptor substrate-1 phosphorylated serine 312 to insulin receptor substrate-phosphorylated tyrosine was higher in PD-MCI compared to PD-NC. αSyn, p-tau 191 and insulin receptor substrate-1 phosphorylated tyrosine contributed to diagnostic classification between groups, suggesting that both αSyn and tau pathology and impaired insulin signaling underlie PD with CI (Blommer et al., 2023). Salivary levels of Aβ-42 were higher in PD than in HCs, αSyn levels lower, while p-tau in PD was not increased in comparison to AD, thus discriminating the two disorders (Sabaei et al., 2023).

Plasma/CSF GFAP may be a valuable prognostic tool for cognitive deterioration. While there was no significant difference in baseline GFAP between PD-NC and MCI groups, higher baseline GFAP levels predicted greater cognitive decline over time in early PD. In addition, it was positively correlated with longitudinal changes not only of CSF αSyn, but also with AD-related biomarkers, namely Aβ-42, t-tau and p-tau (Liu T. et al., 2023). Increased plasma GFAP predicted PD-MCI to dementia conversion, higher than NfL, t-tau and p-tau 181 (Tang et al., 2023). Both serum GFAP and NfL levels were significantly higher in PD-MCI than in HCs and were negatively related with MoCA scores. Therefore, serum GFAP and NfL levels can serve as biomarkers for PD patients at risk for CI (Mao et al., 2023). Although NfL and p-tau 181 plasma levels were significantly increased in PD compared to HCs, only NfL levels were significantly higher in PD-MCI compared to PD-NC at baseline. After follow-up of 4 years, only NfL predicted progression to dementia, while plasma p-tau 181 did not help differentiate PD-MCI and to predict further cognitive deterioration (Pagonabarraga et al., 2022). Recent studies of central nervous system neurochemical profile showed that the great majority (88%) of PD-MCI patients were A–/T–/N– [according to recent AD classification criteria (Jack et al., 2018)]. Among all biomarkers only the ratio between NfL and phosphorylated neurofilament heavy chain was significantly higher in PD-MCI vs. PD-NC (*p* = 0.02). After 2 years, one-third of PD-MCI patients worsened, which was associated with higher baseline levels of NfL, p-tau, and sTREM2 (Paolini Paoletti et al., 2023).

Serum levels of GDNF were associated with multiple other neurotransmitters. Serum GDNF may be involved in the impairment of attention, memory and EF in PD-MCI by acting alone or in conjunction with homovanillic acid and 5-HT that have a correlation with cognition test scores (Liu et al., 2020). PD-MCI patients showed decreased levels of serum GDNF, which was associated with impaired cognitive flexibility, attention performance and inhibitory control. With deterioration of cognitive functions, serum GDNF and homovanillic acid levels decreased and may synergistically participate in development of executive dysfunction in PD (Tong et al., 2023).

Plasma levels of phospholipid were significantly increased in PD-MCI patients compared to PD-NC, and showed a negative correlation with MoCA scores. They reflected membrane injury *in vivo* and might be a marker for the prognosis of cognitive states in PD (Li et al., 2015). Other blood lipid markers that showed increased levels in PD-MCI compared to PD-NC were triglyceride, total cholesterol, high- and low-density lipoprotein cholesterol (HDL-C, LDL-C), and apolipoprotein A1 (ApoA1; Deng et al., 2022). Furthermore, there were strong associations between blood triglyceride, ApoA1, SNCA rs6826785, and PD-MCI with increased risk of PD-MCI (Deng et al., 2023).

Assessment of immune- and inflammatory-related indicators of cognitive dysfunction in PD revealed that Aβ-42, CD4+, CD8+, CD3+, and Treg cell levels were lower in the PD-NC group than in controls while higher than the PD-MCI group. The levels of tau, IL-6, IL-17, and TNF-α in PD-NC were higher than in the HC group but lower in PD-MCI. The combination of p-tau, CD8+, and TNF-α levels was associated with cognitive decline in PD, which can predict cognitive dysfunction in PD (Zhao et al., 2024).

In conclusion, the combination of p-tau 181, NfL, and GFAP in plasma and/or CSF appears currently to be the best marker for PD-MCI and predicting the development of CI in PD. Other combinations are p-tau 181, CD8+, and TNF-α, while inreased p-tau 181 and low Aβ-42 may differentiate PD-MCI from controls.

## 11 Pathophysiological aspects of PD-MCI

LBs in cerebral cortex are present in practically all cases of sporadic PD. Therefore, the distribution of Lewy body pathology (LBP) is possibly one route or pattern of αSyn spreading in early disease stages. LBP involves the brain, extending from olfactory bulb via brainstem, amygdala to the neocortex (Braak et al., 2004). LBP may also start in the enteric system ascending via the vagus nerve to the brain (Borghammer et al., 2021), suggesting that either αSyn aggregation begins in the gut and spreads in a prion-like fashion to the brain or systemic inflammatory processes driven by gastrointestinal dysfunction contribute to the pathogenesis of PD before the development of overt clinical symptoms (Ryman et al., 2023). Immune and inflammatory-related changes may represent another important factor in the pathogenesis of PD, inflammatory markers being associated with cognitive dysfunction in PD (Zhao et al., 2024). However, there is a clear morphological heterogeneity in PD-MCI, similar to that in MCI cases without PD (Markesbery, 2010). It includes a variable combination of LBP and AD-related changes (ADNC), both of which with much lower severity and extent than in fully developed PDD (Jellinger, 2023).

A recent review confirmed the Braak hypothesis of LBP staging from molecular, cell biological (cell-to-cell spread) and organ-level (region-to-region spread) of αSyn pathology (Carceles-Cordon et al., 2023). However, LBP does not always follow the same bottom-up progression from olfactory bulb via brainstem to the cortex, as there may be a multifocal distribution pattern of αSyn pathology (Uchihara and Giasson, 2016). It may involve the NBM, amygdala and locus ceruleus in the prodromal phase of the body-first type of PD (Borghammer et al., 2021). Recent studies provided insight into dysfunctions of dopaminergic, cholinergic and noradrenergic systems that are involved in PD-CI (Ye, 2022). Reduced presynaptic dopamine uptake in the striatum resulted in reduced prefrontal and parietal metabolism (Ekman et al., 2012) and increased signal variability in the SAN, which in turn caused decreased corticostriatal connectivity (Shafiei et al., 2019; Shima et al., 2023). PD-MCI patients compared to PD-NC showed a more widespread degeneration of dopaminergic terminals in the caudate nucleus (Sasikumar and Strafella, 2020), while degeneration of the medial substantia nigra caused dysfunction of striato-frontal and mesocortico-limbic loss (Martínez-Horta and Kulisevsky, 2011). Early cognitive deficits in PD were closely related to the degeneration of the cholinergic forebrain, early degeneration of the NBM and the nucleus of the vertical limb of the diagonal band of Broca, that precede CI in PD (Liu et al., 2018; Ray et al., 2018; Schulz et al., 2018). PD-MCI converters had significantly greater MD of both NBM tracts compared to PD-NC, which was associated with cognitive outcome (psychomotor speed, working memory, delayed recall, and visuospatial function; Crockett et al., 2023). Degeneration of the cholinergic posterior basal forebrain in PD was accompanied by functional cortical changes in acetylcholinesterase activity that was associated with multi-domain cognitive deficits in PD without dementia (Schumacher et al., 2023).

Modern neuroimaging studies documented dysfunction of multiple cortical and subcortical neuronal systems with disruption of essential interconnected brain networks which result from the presence and extension of both LBP and co-morbid ADNC into the limbic and higher cortical association areas. Common mechanisms of PD-MCI and PDD involve not only the aggregation and spreading of αSyn/LBs but also of Aβ and tau in cortical and limbic regions (Masliah et al., 2001; Esteves and Cardoso, 2020). The association of cortical αSyn pathology, Aβ and tau load suggests an interaction of these pathological proteins in the pathogenesis of PD and other neurodegenerative disorders (Clinton et al., 2010; Compta et al., 2011; Jellinger, 2012; Guo et al., 2013; Miller et al., 2022). A potential mechanism for these synergistic effect may be phosphorylation of tau protein induced by αSyn as a posttranslational modification and vice versa (Giasson et al., 2003; Walker et al., 2015; Basheer et al., 2023). Post-mortem studies have confirmed the concomitant protein action, such as the co-aggregation of αSyn, Aβ and tau in PD brains with and without CI (Han and He, 2023; Noguchi-Shinohara and Ono, 2023). It should emphasized that, in general, one of these mechanisms alone may not explain the development of CI in PD, whereas at least two or more molecular mechanisms may concur and contribute to cognitive decline in PD (Irwin et al., 2013; Jellinger, 2022). Recent autopsy studies revealed decreased synaptic density in temporal, cingulate and insular cortices in PD and in the entorhinal and parahippocampal region in PDD, which was associated with higher NfL immunoreactivity, LB density and higher CI scores. In additon, synaptic loss was linked to axonal loss and αSyn burden. These results indicate a relation between synaptic loss in specific brain regions, αSyn burden and CI in PD (Frigerio et al., 2024). Other studies suggest that both αSyn and tau pathologies and impaired insulin signaling underlie CI in PD (Blommer et al., 2023). Human αSyn overexpression in the mesencephalon leads to its increase in the hippocampus inducing altered synaptic transmission and plasticity, with deceased expression of glutamate receptors. This may cause involvement of major neuronal networks leading to memory impairment in PD (Iemolo et al., 2023).

A different pathogenetic model reflecting region-specific changes in gene expression in αSyn inducing a model of early PD appears of interest. In rats, after infusion of human αSyn oligomers (H-αSynOs) into the substantia nigra, the transcription profile in regions involved with MCI were examined. Among more than 17,000 genes expressed in the hippocampus and anterior cingulate cortex, a few were differently expressed (up- or down-regulated) and related with immune functions with decreased expression of CD68 within microglia cells. In contrast, the most significantly enriched terms in the hippocampus were involved in mitochondrial homeostasis, potassium voltage-gated channel, cytoskeleton, and fiber organization, suggesting that the gene expression in the neuronal population was mostly affected in this region in early disease stages. These results show that H-αSynOs trigger a region-specific dysregulation of gene expression in anterior cingulate cortex and hippocampus as pathological substrate of MCI in early PD (Manchinu et al., 2024). The applicability of this interesting rat model to pathology is a matter of further discussion.

In conclusion, cognitive involvement in PD-MCI is likely to arise from a combination of mechanisms causing complex and multilocal cerebral lesions that induce dysfunction of both whole-brain functional networks and dysfunction of several neuromodulator systems, especially loss of cholinergic function. The interplay of pathological accumulation of αSyn as well as tau and Aβ is essential for neurodegeneration inducing these complex pathogenic mechanisms associated with regional synaptic loss, which are responsible for cognitive decline in PD, yet their causative interplay needs further elucidation.

## 12 Management options for MCI in PD

A range of dopaminergic or psychotropic medications, psychotherapeutic techniques, stimulation therapies, cognitive training and rehabilitation methods, and other non-pharmacological treatments have been studied and are used for managing MCI in PD, but appropriate management of CI (and other neuropsychiatric symptoms) is critical for comprehensive PD care (Weintraub et al., 2022). An earlier study suggested that L-dopa might prevent a decline in cognitive function in PD (Ikeda et al., 2017); another one hypothesized that a combined stimulation of both dopamine receptor families with dopamine agonists (rotigotine, carbergoline), L-dopa and pergolide may preserve cognitive function, whereas no differences were found in cognitive function between the basal state and treatment with dopamine agonists alone (Brusa et al., 2013). No significant difference in anticholinergic burden was found between PD-MCI and PD-NC (Sumbul-Sekerci et al., 2022), while a recent review of nine articles stated that there is evidence that rivastigmine is beneficial for rapid eye movement sleep behavior disorder (RBD) and apathy in PD without dementia and may reduce falls, which may be due to improved attention. However, the outcomes of the reviewed studies were heterogenous (Reilly et al., 2024). While anti-hypertensive medications were positively correlated with PD-MCI, anticholinergic drugs burden does not appear to modulate MCI risk in PD (Cicero et al., 2022b). However, MCI in PD patients adversely affects medication management, although cognitive areas predicting success in medication management performance are language, event-based prospective memory and visuospatial functions (Sumbul-Sekerci et al., 2023).

A variety of ketogenic therapies were utilized in the MCI groups including a ketogenic diet, low-carbohydrate or Mediterranean diet with coconut oil supplementation, a diet with a ketogenic medium chain triglyceride supplement or other ketogenic compounds. They showed statistically significant improvements in some, although not all, of cognitive measures, although only after 6 months of adherence that was problematic in most of these studies (Price and Ruppar, 2023). A systemic review of therapeutic ketogenic trials gave evidence for cognitive improvement in individuals with MCI, but the number of studies in PD is very small and further research is requested to optimize the utilization of ketogenic interventions in clinical contexts (Bohnen et al., 2023).

Cognitive rehabilitation has been found to improve specific cognitive deficits in PD, but not all cognitive domains may benefit from this method that should be tailored to patient's specific impairments (Giustiniani et al., 2022a,b). A pilot study on the effects of a new integrated and multidisciplinary cognition program based on mindfulness and reminiscence therapy showed a significant improvement in MoCA memory score and could be effective in PD-MCI patients (Reitano et al., 2023). Cognitive training can increase physical activity possibly due to effects on executive function, but this also needs further investigation in larger samples (Bode et al., 2023).

In conclusion, the effects of medical intervention in PD patients with MCI are controversial, although among anticholinergic drugs, rivastigmine may improve attention, and some cognitive improvement was reported for ketogenic diets and for both cognitive rehabilitation and mindfulness therapy. For most of these methods, the number of trials is too small and further research for their optimization and validation are warranted.

## 13 Conclusions and outlook

PD, a common and heterogeneous neurodegenerative disease, characterized by a combination of motor and non-motor symptoms, is frequently preceded by a period of MCI, affecting multiple cognitive domains, which may or may not progress to dementia or may revert to normal cognition. The clinical manifestations, molecular/biochemical and morphological basis of PD-MCI are heterogeneous, and modern neuroimaging studies revealed widespread changes in cerebral GM and WM, involving multiple brain areas and causing disruption of many critical neuronal networks involved in cognitive, attention and memory functions as well as various neuromodulator systems, lesions which often antedate structural changes. Given the inherent heterogeneity in the clinical presentation, neuropsychology, functional morphology and neuropathology of PD-MCI, diagnostic criteria regarding definition and classification have been established, and modern neuroimaging studies have given insight into the underlying neurobiological mechanism including early metabolic changes. Artificial intelligence on FDG-PET images has identified MCI patients not only in PD but also in other neurodegenerative diseases with 75% sensitivity and 84% specificity (Prats-Climent et al., 2022). The pathogenesis, among others, depends on the extent and severity of LBP and ADNC co-pathologies, mainly involving limbic and subcortical brain areas with less severe extension to the neocortex. A deeper understanding of the essential molecular/biochemical and neurotransmitter changes and in particular of the responsible brain network disorders is required to better understand the relationship of the different types of PD-MCI and their relations with other clinical symptoms. The prospective assessment and validation of MCI and a deeper understanding of the interaction of multiple genetic factors will be achieved by modern fluid biomarkers (e.g., reduced αSyn and Aβ levels, increased p-tau 128, GFAP, and NfL), as well as inflammatory, EEG and diffusion and structural MRI markers. These and other studies will provide a deeper understanding of the pathophysiological processes underlying the timely progress and development of cognitive decline in relation to the neurodegenerative changes in PD-MCI as a basis for the development of effective disease-modifying therapies and preventive measures to slow or halt progression of cognitive impairment in this debilitating disease.

## Author contributions

KJ: Conceptualization, Data curation, Formal analysis, Funding acquisition, Investigation, Methodology, Project administration, Resources, Supervision, Validation, Visualization, Writing—original draft, Writing—review & editing.

## In memoriam

Dedicated to the memory of my friend Gregor K. Wenning who passed away on February 11, 2024.
