# Supplementary material for: Mild cognitive impairment in Parkinson's disease: current view
Source: Front Cognit. 2024 Apr 5;3:1369538. doi: 10.3389/fcogn.2024.1369538 (PMC13281060; doi:10.3389/fcogn.2024.1369538)
Supplement: Supplementary file 1 [file Table_1.doc]

**Table 1 (SUPPLEMENT): Major neuroimaging finding in Parkinson disease with mild cognitive impairment (PD-MCI) versus PD without cognitive impairment (PD-NC) (modified and expanded from Jellinger 2024)**

|  | References |
| --- | --- |
| *General* |  |
| Increased global brain atrophy | Mak et al. 2017 |
| Mild diffuse brain atrophy | Martin et al. 2009; Pereira et al. 2014 |
| Enlargement bilateral temporal, lateral ventricles, atrophy hippocampus Enlargement third ventricle | Apostolova et al. 2012; Gao et al. 2023 |
| *Gray matter atrophy - Cortical structures* |  |
| Medial temporal cortex | Martin et al. 2009; Pereira et al. 2014 |
| Higher rate of cortical thinning in frontal / supplementary motor area, parietal-temporal and occipital cortices | Hanganu et al. 2014; Mak et al. 2015 |
| Prefrontal, limbic lobes, temporal gyrus | Gao et al. 2017 |
| Frontotemporal cortex, parietal and occipital lobes | Weintraub et al. 2011 |
| Orbitofrontal regions, left superior lobule, limbic, fronto-parietal regions | Kunst et al. 2019 |
| Left inferior and orbital frontal gyrus, left anterior insula | Zheng et al. 2019 |
| Prefrontal, medial and lateral temporal cortex | Pletcher et al. 2023 |
| Frontal, temporal, parietal and occipital cortex, striatum | Devignes et al. 2022 |
| Frontal, temporal, parietal cortex, right middle frontal and superior temporal gyrus | Li et al. 2022 |
| Left superior frontal cortex, bilateral entorhinal cortex, precuneus, angular gyrus, and bilateral cerebellum | Donzuso et al. 2021 |
| Anterior cingulate and right parietal lobe | Chen et al. 2016; Hong et al. 2012 |
| Bilateral dorsolateral prefrontal cortex, left insula, angular gyrus, cingulate and right supramarginal gyrus, midcingulate cortex, right hippocampus | Mihaescu et al. 2019 |
| Left inferior and orbital frontal gyrus, left anterior insula | Zheng et al. 2019 |
| Left superior frontal and temporal lobe and left insula | Xu et al. 2016 |
| Superior temporal cortex, lingula, insula, fusiform area | Zhu et al. 2022 |
| Temporal and parietal cortex, amygdala, hippocampus, putamen, cerebellum | Melzer et al. 2012 |
| Medial and superior frontal, inferior temporal, cingulate, supramarginal gyri | Filippi et al. 2020 |
| *Gray matter atrophy - Subcortical structures* |  |
| Entorhinal cortex | Goldman et al. 2012; Jia et al. 2019 |
| Nucleus accumbens | Mak et al. 2014 |
| Nucleus basalis of Meynert | Schulz et al. 2018 |
| Subcortical structures: caudate nucleus, putamen, thalamus, amygdala, nucleus accumbens, NBM, presubiculum | Crowley et al. 2022; Foo et al. 2017 |
| Hippocampal-amygdaloid transition area, CA! region | Becker et al. 2021 |
| Amygdala, hippocampus CA1 and subiculum | Zhang et al. 2023b |
| Amygdala, thalamus | Chen et al. 2016; Schulz et al. 2018 |
| *White matter lesions* |  |
| Higher WM lesion burden periventricular and deep WM | Choi et al. 2010; Huang et al. 2020; Zhao et al. 2023 |
| WMH prefrontal and temporal lobe | Dunet et al. 2019 |
| Involvement corpus callosum, cingulum, frontal, interhemispheric WM | Agosta et al. 2014 |
| Reduced FA and increased mean diffusivity across frontal WM region | Minett et al. 2018 |
| Reduced FA fronto-occipital fascicle | Yu et al. 2023 |
| High WMH burden occipital WM | Carvalho de Abreu et al. 2023 |
| Reduced FA WM corpus callosum | Yu et al. 2023 |
| Decreased FA bilateral frontal and temporal lobes, corpus callosum, cingulum, fornix, longitudinal and fronto-occipital fasciles, bilateral parieto-occipital tract | Pu et al. 2020 |
| Periventricular WM | Scamarcia et al. 2022 |
| Reduction corpus callosum, cingulum, longitudinal fascicles | Liao et al. 2023 |
| WM microstructure lesions | Melzer et al. 2013; Sarasso et al. 2021 |
| Lower density prefrontal region, cingulum bundle, thalamofrontal tracts | Zhang et al. 2023a |
| Microstructural changes in anterior olfactory structures | Stewart et al. 2023 |
| Decreased FA in fronto-occipital fascicles | Kübler et al. 2023 |
| Decreased FA in superior longitudinal fascicles | Yu et al. 2023 |
| *Reduced glucose metabolism* |  |
| Frontal lobe > parietal and occipital areas | Garcia-Garcia et al. 2012 |
| Atrophy angular gyrus, orbital, anterior frontal and occipital lobe | González-Redondo et al. 2014 |
| Parietal, cingulate cortex, precuneus, hippocamous, occipital lobes | Bohnen et al. 2011; Homenko et al. 2017 |
| Frontal and posterior cortex, bilateral occipital lobe | Zhihui et al. 2023 |
| *Brain network dysfunctions* |  |
| Loss of FC in SAN without structural changes | Aracil-Bolaños et al. 2019 |
| Dysfunction of sensorimotor, executive and neurocognitive networks | Lang et al. 2020 |
| Reduced FC between striatal network, DMN, central executive and SAN | Lang et al. 2020 |
| Increased FC betwen left hippocampus and right cerebellar hemisphere | Lang et al. 2020 |
| Reduced FC DMN between hippocampus and inferior frontal cortex, posterior cingulate cortex and posterior parietal lobuls, anterior temporal lobe and inferior frontal gyrus, middle frontal and middle temporal gyrus, between DMN and precentral middle temporal gyrus, insula, anterior parietal lobule and middle frontal cortex | Hou et al. 2016 |
| Reduced FC between bilateral frontoparietal network, ventrolateral and dorsolateral prefrontal cortex, dorsolateral prefrontal cortex, inferior frontal gyrus | Liu et al. 2023 |
| Abnormal intrinsic FC within DMN, ECN and SAN, decoupling left and right FPN | De Micco et al. 2023 |
| Decreased FC in DMN and FPN | Amboni et al. 2015 |
| Disrupted FC in SMN, DMN and FPN, DMN-CN | Suo et al. 2021b |
| Disrupted DMN and cerebellar network | Suo et al. 2021a |
| Reduced FC multi-connect networks in bilatera orbitofrontal lobe | Wang et al. 2020 |
| Reduced FC between mediodorsal thalamus and paracingulate cortex Increased FC mediodorsal thalamus and posterior cingulate cortex | Owens-Walton et al. 2021 |
| Disrupted network between bilateral superior medial frontal cortex and anterior/middle cingulate cortex | Maier et al. 2023 |
| Increased FC between caudate head to left hippocampus and right cerebellum | Maier et al. 2023 |
| Decreased FC between right caudate head and anterior cingulate cortex, precuneus and left supramarginal gyrus | Lang et al. 2020 |
| Disrupted FC between frontal and posterior cortical regions | Chung et al. 2022 |
| Decreased FC in SMN and executive processing networks | Mihaescu et al. 2019 |
| Decreased FC left superior temporal/fusiform -> right insula | Zhu et al. 2022 |
| Interrupted network linking temporal-parietal-occipital lobes | Chu et al. 2023 |
| Decreased FC DAN, VAN, FPN, SMN | Delgado-Alvarado et al. 2023 |
| Decreased FC between frontostriatal and posterior cortical areas | Devignes et al. 2022 |
| Disrupted FC between frontal cortex and posterior cortical regions | Chen et al. 2022; Chung et al. 2019 |
| Reduced FC between right frontal and bilateral parietal areas Decreased FC between substantia innominata and frontal area | Kim et al. 2017 |
| Disrupted FC between frontoparietal-visual-sensorimotor and subcortical networks | Shang et al. 2023 |
| Increased amplitude of low-frequency intrinsic fluctuations and regional homogeneity in the DMN | Guo et al. 2021; Harrington et al. 2017 |
| *Other changes* |  |
| High free water fraction in caudate nucleus, bilateral NBM | Crowley et al. 2022 |
| Mild cortical Aβ binding in cingulate and middle temporal gyri, not significantly different from PD with normal cognition and age-matched healthy controls | Huang et al. 2023; Melzer et al. 2019; Palermo et al. 2019; Petrou et al. 2012 |
| Rare and minimal cortical tau deposition | Coughlin et al. 2020; Gomperts et al. 2016; Kantarci et al. 2017 |
| Reduced neuromelanin-sensitive PET scan signal in substantia niga and locus ceruleus | Marquie et al. 2017 |

DMN: default mode network; FA: fractional anisotrophy; FC: functional connectivity; FPN: frontoparietal network; SAN: salience network; DAN: dorsal attention network; VAN: ventral attention network; CN: cerebellar network; ECN: executive function network

References

Agosta F, Canu E, Stefanova E, Sarro L, Tomic A, Špica V, Comi G, Kostic VS, Filippi M (2014) Mild cognitive impairment in Parkinson's disease is associated with a distributed pattern of brain white matter damage. Hum Brain Mapp 35:1921-1929

Amboni M, Tessitore A, Esposito F, Santangelo G, Picillo M, Vitale C, Giordano A, Erro R, de Micco R, Corbo D, Tedeschi G, Barone P (2015) Resting-state functional connectivity associated with mild cognitive impairment in Parkinson's disease. J Neurol 262:425-434

Apostolova L, Alves G, Hwang KS, Babakchanian S, Bronnick KS, Larsen JP, Thompson PM, Chou YY, Tysnes OB, Vefring HK, Beyer MK (2012) Hippocampal and ventricular changes in Parkinson's disease mild cognitive impairment. Neurobiol Aging 33:2113-2124

Aracil-Bolaños I, Sampedro F, Marín-Lahoz J, Horta-Barba A, Martínez-Horta S, Botí M, Pérez-Pérez J, Bejr-Kasem H, Pascual-Sedano B, Campolongo A, Izquierdo C, Gironell A, Gómez-Ansón B, Kulisevsky J, Pagonabarraga J (2019) A divergent breakdown of neurocognitive networks in Parkinson's disease mild cognitive impairment. Hum Brain Mapp 40:3233-3242

Becker S, Granert O, Timmers M, Pilotto A, Van Nueten L, Roeben B, Salvadore G, Galpern WR, Streffer J, Scheffler K, Maetzler W, Berg D, Liepelt-Scarfone I (2021) Association of hippocampal subfields, CSF biomarkers, and cognition in patients with Parkinson disease without dementia. Neurology 96:e904-e915

Bohnen NI, Koeppe RA, Minoshima S, Giordani B, Albin RL, Frey KA, Kuhl DE (2011) Cerebral glucose metabolic features of Parkinson disease and incident dementia: longitudinal study. J Nucl Med 52:848-855

Carvalho de Abreu DC, Pieruccini-Faria F, Sarquis-Adamson Y, Black A, Fraser J, Van Ooteghem K, Cornish B, Grimes D, Jog M, Masellis M, Steeves T, Nanayakkara N, Ramirez J, Scott C, Holmes M, Ozzoude M, Berezuk C, Symons S, Mohammad Hassan Haddad S, Arnott SR, Binns M, Strother S, Beaton D, Sunderland K, Theyers A, Tan B, Zamyadi M, Levine B, Orange JB, Roberts AC, Lou W, Sujanthan S, Breen DP, Marras C, Kwan D, Adamo S, Peltsch A, Troyer AK, Black SE, McLaughlin PM, Lang AE, McIlroy W, Bartha R, Montero-Odasso M (2023) White matter hyperintensity burden predicts cognitive but not motor decline in Parkinson's disease: results from the Ontario Neurodegenerative Diseases Research Initiative. Eur J Neurol 30:920-933

Chen FX, Kang DZ, Chen FY, Liu Y, Wu G, Li X, Yu LH, Lin YX, Lin ZY (2016) Gray matter atrophy associated with mild cognitive impairment in Parkinson's disease. Neurosci Lett 617:160-165

Chen H, Wan H, Zhang M, Wardlaw JM, Feng T, Wang Y (2022) Perivascular space in Parkinson's disease: Association with CSF amyloid/tau and cognitive decline. Parkinsonism Relat Disord 95:70-76

Choi SA, Evidente VG, Caviness JN, Shill HA, Sabbagh MN, Connor DJ, Hentz JG, Adler CH, Beach TG (2010) Are there differences in cerebral white matter lesion burdens between Parkinson's disease patients with or without dementia? (Correspondence). Acta Neuropathol 119:147-149

Chu C, Zhang Z, Wang J, Wang L, Shen X, Bai L, Li Z, Dong M, Liu C, Yi G, Zhu X (2023) Evolution of brain network dynamics in early Parkinson's disease with mild cognitive impairment. Cogn Neurodyn 17:681-694

Chung SJ, Yoo HS, Lee YH, Lee HS, Ye BS, Sohn YH, Kwon H, Lee PH (2019) Frontal atrophy as a marker for dementia conversion in Parkinson's disease with mild cognitive impairment. Hum Brain Mapp 40:3784-3794

Chung SJ, Kim YJ, Jung JH, Lee HS, Ye BS, Sohn YH, Jeong Y, Lee PH (2022) Association between white matter connectivity and early dementia in patients with Parkinson disease. Neurology 98:e1846-e1856

Coughlin DG, Phillips JS, Roll E, Peterson C, Lobrovich R, Rascovsky K, Ungrady M, Wolk DA, Das S, Weintraub D, Lee EB, Trojanowski JQ, Shaw LM, Vaishnavi S, Siderowf A, Nasrallah IM, Irwin DJ, McMillan CT (2020) Multimodal in vivo and postmortem assessments of tau in Lewy body disorders. Neurobiol Aging 96:137-147

Crowley SJ, Amin M, Tanner JJ, Ding M, Mareci TA, Price CC (2022) Free water fraction predicts cognitive decline for individuals with idiopathic Parkinson's disease. Parkinsonism Relat Disord 104:72-77

De Micco R, Piramide N, Di Nardo F, Siciliano M, Cirillo M, Russo A, Silvestro M, Tedeschi G, Esposito F, Tessitore A (2023) Resting-state network connectivity changes in drug-naive Parkinson's disease patients with probable REM sleep behavior disorder. J Neural Transm 130:43-51

Delgado-Alvarado M, Ferrer-Gallardo VJ, Paz-Alonso PM, Caballero-Gaudes C, Rodríguez-Oroz MC (2023) Interactions between functional networks in Parkinson's disease mild cognitive impairment. Sci Rep 13:20162

Devignes Q, Lopes R, Dujardin K (2022) Neuroimaging outcomes associated with mild cognitive impairment subtypes in Parkinson's disease: A systematic review. Parkinsonism Relat Disord 95:122-137

Donzuso G, Monastero R, Cicero CE, Luca A, Mostile G, Giuliano L, Baschi R, Caccamo M, Gagliardo C, Palmucci S, Zappia M, Nicoletti A (2021) Neuroanatomical changes in early Parkinson's disease with mild cognitive impairment: a VBM study; the Parkinson's Disease Cognitive Impairment Study (PaCoS). Neurol Sci 42:3723-3731

Dunet V, Fartaria MJ, Deverdun J, Le Bars E, Maury F, Castelnovo G, Kober T, Cuadra MB, Geny C, Marechal B, de Champfleur NM (2019) Episodic memory decline in Parkinson' s disease: relation with white matter hyperintense lesions and influence of quantification method. Brain Imaging Behav 13:810-818

Filippi M, Canu E, Donzuso G, Stojkovic T, Basaia S, Stankovic I, Tomic A, Markovic V, Petrovic I, Stefanova E, Kostic VS, Agosta F (2020) Tracking cortical changes throughout cognitive decline in Parkinson's disease. Mov Disord 35:1987-1998

Foo H, Mak E, Yong TT, Wen MC, Chander RJ, Au WL, Sitoh YY, Tan LC, Kandiah N (2017) Progression of subcortical atrophy in mild Parkinson's disease and its impact on cognition. Eur J Neurol 24:341-348

Gao HL, Qu Y, Chen SC, Yang QM, Li JY, Tao AY, Mao ZJ, Xue Z (2023) Third ventricular width by transcranial sonography is associated with cognitive impairment in Parkinson's disease. CNS Neurosci Ther 30:e14360

Gao Y, Nie K, Huang B, Mei M, Guo M, Xie S, Huang Z, Wang L, Zhao J, Zhang Y (2017) Changes of brain structure in Parkinson's disease patients with mild cognitive impairment analyzed via VBM technology. Neurosci Lett 658:121-132

Garcia-Garcia D, Clavero P, Gasca Salas C, Lamet I, Arbizu J, Gonzalez-Redondo R, Obeso JA, Rodriguez-Oroz MC (2012) Posterior parietooccipital hypometabolism may differentiate mild cognitive impairment from dementia in Parkinson's disease. Eur J Nucl Med Mol Imaging 39:1767-1777

Goldman JG, Stebbins GT, Bernard B, Stoub TR, Goetz CG, deToledo-Morrell L (2012) Entorhinal cortex atrophy differentiates Parkinson's disease patients with and without dementia. Mov Disord 27:727-734

Gomperts SN, Locascio JJ, Makaretz SJ, Schultz A, Caso C, Vasdev N, Sperling R, Growdon JH, Dickerson BC, Johnson K (2016) Tau positron emission tomographic imaging in the Lewy body diseases. JAMA Neurol 73:1334-1341

González-Redondo R, García-García D, Clavero P, Gasca-Salas C, García-Eulate R, Zubieta JL, Arbizu J, Obeso JA, Rodríguez-Oroz MC (2014) Grey matter hypometabolism and atrophy in Parkinson's disease with cognitive impairment: a two-step process. Brain 137:2356-2367

Guo W, Jin W, Li N, Gao J, Wang J, Chang Y, Yin K, Chen Y, Zhang S, Wang T (2021) Brain activity alterations in patients with Parkinson's disease with cognitive impairment based on resting-state functional MRI. Neurosci Lett 747:135672

Hanganu A, Bedetti C, Degroot C, Mejia-Constain B, Lafontaine AL, Soland V, Chouinard S, Bruneau MA, Mellah S, Belleville S, Monchi O (2014) Mild cognitive impairment is linked with faster rate of cortical thinning in patients with Parkinson's disease longitudinally. Brain 137:1120-1129

Harrington DL, Shen Q, Castillo GN, Filoteo JV, Litvan I, Takahashi C, French C (2017) Aberrant intrinsic activity and connectivity in cognitively normal Parkinson's disease. Front Aging Neurosci 9:197

Homenko JG, Susin DS, Kataeva GV, Irishina JA, Zavolokov IG (2017) [Characteristics of cerebral glucose metabolism in patients with cognitive impairment in Parkinson's disease]. Zh Nevrol Psikhiatr Im S S Korsakova 117:46-51

Hong JY, Lee JE, Sohn YH, Lee PH (2012) Neurocognitive and atrophic patterns in Parkinson's disease based on subjective memory complaints. J Neurol 259:1706-1712

Hou Y, Yang J, Luo C, Song W, Ou R, Liu W, Gong Q, Shang H (2016) Dysfunction of the default mode network in drug-naive Parkinson's disease with mild cognitive impairments: a resting-state fMRI study. Front Aging Neurosci 8:247

Huang CC, Chen PH, Tsai CC, Chiang HF, Hsieh CC, Chen TL, Liao WH, Chen YL, Wang JJ (2023) Diffusion and structural MRI as potential biomarkers in people with Parkinson's disease and cognitive impairment. Eur Radiol 34:126-135

Huang X, Wen MC, Ng SY, Hartono S, Chia NS, Choi X, Tay KY, Au WL, Chan LL, Tan EK, Tan LC (2020) Periventricular white matter hyperintensity burden and cognitive impairment in early Parkinson's disease. Eur J Neurol 27:959-966

Jellinger KA (2024) Pathobiology of cognitive impairment in Parkinson disease: challenges and outlooks. Int J Mol Sci 25:494. https://doi.org/410.3390/ijms25010498

Jia X, Li Y, Li K, Liang P, Fu X (2019) Precuneus dysfunction in Parkinson's disease with mild cognitive impairment. Front Aging Neurosci 10:427

Kantarci K, Lowe VJ, Boeve BF, Senjem ML, Tosakulwong N, Lesnick TG, Spychalla AJ, Gunter JL, Fields JA, Graff-Radford J, Ferman TJ, Jones DT, Murray ME, Knopman DS, Jack CR, Jr., Petersen RC (2017) AV-1451 tau and beta-amyloid positron emission tomography imaging in dementia with Lewy bodies. Ann Neurol 81:58-67

Kim I, Shin NY, Yunjin B, Hyu Lee P, Lee SK, Mee Lim S (2017) Early-onset mild cognitive impairment in Parkinson's disease: Altered corticopetal cholinergic network. Sci Rep 7:2381

Kübler D, Kobylecki C, McDonald KR, Anton-Rodriguez JM, Herholz K, Carter SF, Hinz R, Thompson JC, Al-Fatly B, Gerhard A (2023) Structural and metabolic correlates of neuropsychological profiles in multiple system atrophy and Parkinson's disease. Parkinsonism Relat Disord 107:105277

Kunst J, Marecek R, Klobusiakova P, Balazova Z, Anderkova L, Nemcova-Elfmarkova N, Rektorova I (2019) Patterns of grey matter atrophy at different stages of Parkinson's and Alzheimer's diseases and relation to cognition. Brain Topogr 32:142-160

Lang S, Yoon EJ, Kibreab M, Kathol I, Cheetham J, Hammer T, Sarna J, Ismail Z, Monchi O (2020) Mild behavioral impairment in Parkinson's disease is associated with altered corticostriatal connectivity. Neuroimage Clin 26:102252

Li L, Ji B, Zhao T, Cui X, Chen J, Wang Z (2022) The structural changes of gray matter in Parkinson disease patients with mild cognitive impairments. PLoS One 17:e0269787

Liao TW, Wang JJ, Tsai CC, Wang PN, Chen YL, Wu YM, Wu YR (2023) A fixel-based analysis of white matter reductions early detects Parkinson disease with mild cognitive impairment. Biomed J:100678

Liu J, Zou X, Gu J, Yu Q, Dong Z, Zuo H, Chen X, Du X, Zou D, Han Y, Peng J, Cheng O (2023) Altered connectivity in the cognitive control-related prefrontal cortex in Parkinson's disease with rapid eye movement sleep behavior disorder. Brain Imaging Behav 17:702-714

Maier F, Greuel A, Hoock M, Kaur R, Tahmasian M, Schwartz F, Csoti I, Jessen F, Drzezga A, van Eimeren T, Timmermann L, Eggers C (2023) Impaired self-awareness of cognitive deficits in Parkinson's disease relates to cingulate cortex dysfunction. Psychol Med 53:1244-1253

Mak E, Bergsland N, Dwyer MG, Zivadinov R, Kandiah N (2014) Subcortical atrophy is associated with cognitive impairment in mild Parkinson disease: a combined investigation of volumetric changes, cortical thickness, and vertex-based shape analysis. AJNR Am J Neuroradiol 35:2257-2264

Mak E, Su L, Williams GB, Firbank MJ, Lawson RA, Yarnall AJ, Duncan GW, Owen AM, Khoo TK, Brooks DJ, Rowe JB, Barker RA, Burn DJ, O'Brien JT (2015) Baseline and longitudinal grey matter changes in newly diagnosed Parkinson's disease: ICICLE-PD study. Brain 138:2974-2986

Mak E, Su L, Williams GB, Firbank MJ, Lawson RA, Yarnall AJ, Duncan GW, Mollenhauer B, Owen AM, Khoo TK, Brooks DJ, Rowe JB, Barker RA, Burn DJ, O'Brien JT (2017) Longitudinal whole-brain atrophy and ventricular enlargement in nondemented Parkinson's disease. Neurobiol Aging 55:78-90

Marquie M, Verwer EE, Meltzer AC, Kim SJW, Aguero C, Gonzalez J, Makaretz SJ, Siao Tick Chong M, Ramanan P, Amaral AC, Normandin MD, Vanderburg CR, Gomperts SN, Johnson KA, Frosch MP, Gomez-Isla T (2017) Lessons learned about [F-18]-AV-1451 off-target binding from an autopsy-confirmed Parkinson's case. Acta Neuropathol Commun 5:75

Martin WR, Wieler M, Gee M, Camicioli R (2009) Temporal lobe changes in early, untreated Parkinson's disease. Mov Disord 24:1949-1954

Melzer TR, Watts R, MacAskill MR, Pitcher TL, Livingston L, Keenan RJ, Dalrymple-Alford JC, Anderson TJ (2012) Grey matter atrophy in cognitively impaired Parkinson's disease. J Neurol Neurosurg Psychiatry 83:188-194

Melzer TR, Watts R, MacAskill MR, Pitcher TL, Livingston L, Keenan RJ, Dalrymple-Alford JC, Anderson TJ (2013) White matter microstructure deteriorates across cognitive stages in Parkinson disease. Neurology 80:1841-1849

Melzer TR, Stark MR, Keenan RJ, Myall DJ, MacAskill MR, Pitcher TL, Livingston L, Grenfell S, Horne KL, Young BN, Pascoe MJ, Almuqbel MM, Wang J, Marsh SH, Miller DH, Dalrymple-Alford JC, Anderson TJ (2019) Beta amyloid deposition is not associated with cognitive impairment in Parkinson's disease. Front Neurol 10:391

Mihaescu AS, Masellis M, Graff-Guerrero A, Kim J, Criaud M, Cho SS, Ghadery C, Valli M, Strafella AP (2019) Brain degeneration in Parkinson's disease patients with cognitive decline: a coordinate-based meta-analysis. Brain Imaging Behav 13:1021-1034

Minett T, Su L, Mak E, Williams G, Firbank M, Lawson RA, Yarnall AJ, Duncan GW, Owen AM, Khoo TK, Brooks DJ, Rowe JB, Barker RA, Burn D, O'Brien JT (2018) Longitudinal diffusion tensor imaging changes in early Parkinson's disease: ICICLE-PD study. J Neurol 265:1528-1539

Owens-Walton C, Jakabek D, Power BD, Walterfang M, Hall S, van Westen D, Looi JCL, Shaw M, Hansson O (2021) Structural and functional neuroimaging changes associated with cognitive impairment and dementia in Parkinson's disease. Psychiatry Res Neuroimaging 312:111273

Palermo G, Tommasini L, Aghakhanyan G, Frosini D, Giuntini M, Tognoni G, Bonuccelli U, Volterrani D, Ceravolo R (2019) Clinical correlates of cerebral amyloid deposition in Parkinson's disease dementia: evidence from a PET study. J Alzheimers Dis 70:597-609

Pereira JB, Svenningsson P, Weintraub D, Brønnick K, Lebedev A, Westman E, Aarsland D (2014) Initial cognitive decline is associated with cortical thinning in early Parkinson disease. Neurology 82:2017-2025

Petrou M, Bohnen NI, Muller ML, Koeppe RA, Albin RL, Frey KA (2012) Abeta-amyloid deposition in patients with Parkinson disease at risk for development of dementia. Neurology 79:1161-1167

Pletcher C, Dabbs K, Barzgari A, Pozorski V, Haebig M, Wey S, Krislov S, Theisen F, Okonkwo O, Cary P, Oh J, Illingworth C, Wakely M, Law L, Gallagher CL (2023) Cerebral cortical thickness and cognitive decline in Parkinson's disease. Cereb Cortex Commun 4:tgac044

Pu W, Shen X, Huang M, Li Z, Zeng X, Wang R, Shen G, Yu H (2020) Assessment of white matter lesions in Parkinson's disease: voxel-based analysis and tract-based spatial statistics analysis of Parkinson's disease with mild cognitive impairment. Curr Neurovasc Res 17:480-486

Sarasso E, Agosta F, Piramide N, Filippi M (2021) Progression of grey and white matter brain damage in Parkinson's disease: a critical review of structural MRI literature. J Neurol 268:3144-3179

Scamarcia PG, Agosta F, Spinelli EG, Basaia S, Stojkovic T, Stankovic I, Sarasso E, Canu E, Markovic V, Petrovic I, Stefanova E, Pagani E, Kostic VS, Filippi M (2022) Longitudinal white matter damage evolution in Parkinson's disease. Mov Disord 37:315-324

Schulz J, Pagano G, Fernández Bonfante JA, Wilson H, Politis M (2018) Nucleus basalis of Meynert degeneration precedes and predicts cognitive impairment in Parkinson's disease. Brain 141:1501-1516

Shang S, Zhu S, Wu J, Xu Y, Chen L, Dou W, Yin X, Chen YC, Shen D, Ye J (2023) Topological disruption of high-order functional networks in cognitively preserved Parkinson's disease. CNS Neurosci Ther 29:566-576

Stewart SA, Pimer L, Fisk JD, Rusak B, Leslie RA, Eskes G, Schoffer K, McKelvey JR, Rolheiser T, Khan MN, Robertson H, Good KP (2023) Olfactory function and diffusion tensor imaging as markers of mild cognitive impairment in early stages of Parkinson's disease. Clin EEG Neurosci 54:91-97

Suo X, Lei D, Li N, Li J, Peng J, Li W, Yang J, Qin K, Kemp GJ, Peng R, Gong Q (2021a) Topologically convergent and divergent morphological gray matter networks in early-stage Parkinson's disease with and without mild cognitive impairment. Hum Brain Mapp 42:5101-5112

Suo X, Lei D, Li N, Li W, Kemp GJ, Sweeney JA, Peng R, Gong Q (2021b) Disrupted morphological grey matter networks in early-stage Parkinson's disease. Brain Struct Funct 226:1389-1403

Wang W, Mei M, Gao Y, Huang B, Qiu Y, Zhang Y, Wang L, Zhao J, Huang Z, Nie K (2020) Changes of brain structural network connection in Parkinson's disease patients with mild cognitive dysfunction: a study based on diffusion tensor imaging. J Neurol 267:933-943

Weintraub D, Doshi J, Koka D, Davatzikos C, Siderowf AD, Duda JE, Wolk DA, Moberg PJ, Xie SX, Clark CM (2011) Neurodegeneration across stages of cognitive decline in Parkinson disease. Arch Neurol 68:1562-1568

Xu Y, Yang J, Hu X, Shang H (2016) Voxel-based meta-analysis of gray matter volume reductions associated with cognitive impairment in Parkinson's disease. J Neurol 263:1178-1187

Yu Z, Pang H, Yu H, Wu Z, Ding Z, Fan G (2023) Segmental disturbance of white matter microstructure in predicting mild cognitive impairment in idiopathic Parkinson's disease: An individualized study based on automated fiber quantification tractography. Parkinsonism Relat Disord 115:105802

Zhang C, Yuan Y, Sang T, Yu L, Yu Y, Liu X, Zhou W, Zeng Q, Wang J, Peng G, Feng Y (2023a) Local white matter abnormalities in Parkinson's disease with mild cognitive impairment: Assessed with neurite orientation dispersion and density imaging. J Neurosci Res 101:1154-1169

Zhang L, Zhang P, Dong Q, Zhao Z, Zheng W, Zhang J, Hu X, Yao Z, Hu B (2023b) Fine-grained features characterize hippocampal and amygdaloid change pattern in Parkinson's disease and discriminate cognitive-deficit subtype. CNS Neurosci Ther

Zhao W, Cheng B, Zhu T, Cui Y, Shen Y, Fu X, Li M, Feng Y, Zhang S (2023) Effects of white matter hyperintensity on cognitive function in PD patients: a meta-analysis. Front Neurol 14:1203311

Zheng D, Chen C, Song W, Yi Z, Zhao P, Zhong J, Dai Z, Shi H, Pan P (2019) Regional gray matter reductions associated with mild cognitive impairment in Parkinson's disease: A meta-analysis of voxel-based morphometry studies. Behav Brain Res 371:111973

Zhihui S, Yinghua L, Hongguang Z, Yuyin D, Xiaoxiao D, Lulu G, Yi L, Kangli F, Ying Z (2023) Correlation analysis between (18)F-fluorodeoxyglucose positron emission tomography and cognitive function in first diagnosed Parkinson's disease patients. Front Neurol 14:1195576

Zhu Y, Yang B, Zhou C, Gao C, Hu Y, Yin WF, Yin K, Jiang G, Ren H, Pang A, Yang X (2022) Cortical atrophy is associated with cognitive impairment in Parkinson's disease: a combined analysis of cortical thickness and functional connectivity. Brain Imaging Behav 16:2586-2600
